# Dual Neuroprotective and Nephroprotective Effects of *Mucuna pruriens*, *Moringa oleifera*, and *Silybum marianum* (Milk Thistle) via Modulation of PI3K/AKT/mTOR and Nrf2/NF-κB Pathways in a Murine Comorbid PD–AKI Model

**DOI:** 10.3390/ijms27094021

**Published:** 2026-04-30

**Authors:** Iman Al Housseini, Hoda Dakdouk, Hadi El Natour, Jamilah Borjac

**Affiliations:** 1 Department of Biological Sciences, Faculty of Science, Beirut Arab University, Debbieh 11-5020, Lebanon; iman_alhousseini_05@hotmail.com (I.A.H.); hodadakdouk1461@gmail.com (H.D.); 2 Department of Medicine, Faculty of Medicine, Beirut Arab University, Debbieh 11-5020, Lebanon; hme077@student.bau.edu.lb

**Keywords:** Parkinson’s disease, acute kidney injury, oxidative stress, inflammation, behavioral outcomes

## Abstract

Parkinson’s disease (PD) and acute kidney injury (AKI) are two conditions with increasing prevalence and severe systemic complications and consequences. This research examines the combined neuroprotective and nephroprotective properties of three medicinal plants, *Mucuna pruriens* (Muc), *Moringa oleifera* (Mor), and *Silybum marianum* (SM), in a murine model of PD, AKI, and their comorbid state (PD–AKI), highlighting the role of the PI3K/AKT/mTOR and Nrf2/NF-κB signaling pathways. The mice were grouped as PD, AKI, or PD-AKI, and with or without the herbal pre-treatment, along with their respective controls. Motor impairments were assessed using the rotarod and pole climb assays. Biochemical indicators of renal function, oxidative stress markers, and inflammatory cytokines were quantified in kidney and brain tissues. Assessment of *Nrf2*, *NF-κB*, *PI3K*, *AKT*, and *mTOR* expression levels was performed using qRT-PCR. The AKI groups had significant renal impairment (4-fold increase in creatinine and 7.5-fold increase in BUN), oxidative stress (~5.5-fold increase), and increased cytokine levels (~1.5-fold increase), with downregulation of the PI3K/AKT/mTOR (~2-fold decrease) and Nrf2 signaling pathways (~1.8-fold decrease), alongside upregulation of NF-κB (~2.5-fold increase). The PD and PD-AKI groups exhibited significant neuroinflammation (~1.5-fold increase) and redox imbalance (~6-fold increase) in brain tissue, accompanied by motor impairments (1.6 to 4.6-fold decrease). Pre-treatment with Muc, Mor, and SM significantly ameliorated renal impairments (3.5-fold decrease in creatinine and ~5-fold decrease in BUN) and neurological deficits. These findings establish Muc, Mor, and SM extracts as potent, multi-target interventions capable of disrupting the feed–forward cycle of neuro-renal damage.

## 1. Introduction

Parkinson’s disease (PD) is a progressive brain disorder characterized by the loss of dopaminergic neurons in the substantia nigra [1], the accumulation of α-synuclein aggregates [2], motor impairments, cognitive decline, and progressive deterioration of overall health [3]. Although PD is considered a brain disorder, recent findings have indicated that it also impacts other parts of the body, such as the kidneys [4], possibly impacting the prognosis and the severity of the condition.

Acute kidney injury (AKI), characterized by sudden deterioration in renal function and linked to oxidative stress, inflammation, and tubular damage, often occurs in individuals with chronic conditions, such as PD [4,5,6]. Patients suffering from both PD and AKI present a distinct clinical dilemma since one disorder may aggravate the pathophysiology of the other [6]. AKI acts as a significant, modifiable risk factor for PD. It can trigger systemic inflammation, oxidative stress, disruption of the blood–brain barrier, and accumulation of neuronal proteins, such as α-synuclein [5]. These factors can undermine central nervous system integrity and increase the vulnerability of dopaminergic neurons [4]. In contrast, PD-associated autonomic dysfunction and inflammatory responses may increase the vulnerability to renal damage [7]. Although growing evidence supports the existence of a brain–kidney axis, the bidirectional influence of cerebral and renal health remains insufficiently understood [7,8,9,10].

There is a growing interest in using plant-derived remedies as ways to prevent or support the treatment of long-term diseases characterized by oxidative stress and inflammation [11,12,13]. *Mucuna pruriens* (Muc) is recognized for its substantial L-3,4-dihydroxyphenylalanine (L-DOPA) levels, predominantly concentrated in its seeds, making up 3 to 7% of the plant’s dry weight [14]. This could be a potential natural treatment for PD, as L-DOPA is the direct precursor to dopamine that possesses neuroprotective properties [15,16]. Muc has demonstrated efficacy in PD clinical trials. A recent systematic analysis demonstrated significant potential in enhancing motor symptoms and diminishing therapy-related problems in people with PD [17]. Nonetheless, existing clinical evidence is insufficient, necessitating additional studies and investigations to validate its efficacy, safety, and underlying molecular mechanism. In PD models, Muc was shown to significantly minimize arsenic-induced nephrotoxicity by inhibiting reactive oxygen species (ROS)-mediated renal tubular injury and maintaining glomerular filtration functions [18].

*Moringa oleifera* (Mor), the ‘miracle tree,’ contains a variety of natural compounds, like flavonoids, phenolic acids, and many other beneficial substances [19], that collectively have significant antioxidant, anti-inflammatory [20,21,22], and neuroprotective properties [23,24]. Preclinical investigations have repeatedly shown Mor’s effectiveness in safeguarding neuronal and renal tissues from toxic damage by modifying oxidative stress indicators, inhibiting lipid peroxidation, and increasing glutathione levels [25]. Studies have demonstrated that it enhances the histological architecture of the kidney [26,27] and brain in various types of chemically induced damage [28,29]. *Silybum marianum* (SM), commonly known as Milk Thistle, is a medicinal herb primarily known for its active compound, silymarin [30,31]. Silymarin acts as a free radical scavenger [32] and regulates pathways associated with inflammation [33], fibrosis [34], and apoptosis [35]. In studies on brain health, it protects against harmful brain signals, oxidative damage [33,36], and behavioral impairments [33,37]. In kidney studies, SM offers protective effects by inhibiting the absorption of nephrotoxic substances and downregulating apoptosis [38] and provides protection against drug-induced nephrotoxicity [39].

Although the individual protective effects of Muc, Mor, and SM have been documented in separate models of neurological or renal injury, no previous study has systematically evaluated their prophylactic efficacy in the context of concurrent PD-AKI conditions. Both PD and AKI share common pathological mechanisms, including oxidative stress, mitochondrial dysfunction, and inflammation, and have been more frequently discussed in the literature as potentially interconnected through a proposed brain–kidney axis. Therefore, investigating a combined phytotherapeutic approach may provide a more comprehensive strategy to target these overlapping pathways.

Emerging evidence indicates a “brain–kidney axis,” in which renal dysfunction may worsen neurological decline and vice versa. The renal dysfunction leads to neurological decline due to uremic toxin accumulation, systemic inflammation, oxidative stress, and disruption of the blood–brain barrier [10,40,41]. On the other hand, neurodegenerative processes and central nervous system dysregulation may exacerbate renal injury through sympathetic nervous system activation, neuroinflammation, and altered hemodynamic control [42,43]. However, these interactions are still not well understood and are not directly investigated in the present study. Nevertheless, limited research has experimentally modeled this dual pathology or investigated therapeutic interventions aimed at both organs concurrently.

Thus, this research establishes a novel dual-insult mouse model integrating rotenone (PD) and acetaminophen (APAP)-induced AKI. We investigate inter-organ crosstalk mediated by oxidative stress and inflammation, focusing specifically on the PI3K/AKT/mTOR survival pathway as a shared molecular bridge, and assess the efficacy of natural herbs in alleviating behavioral impairments and the dual pathological consequences.

## 2. Results

### 2.1. Renal Function Parameters: Creatinine and BUN

**Figure 1:**
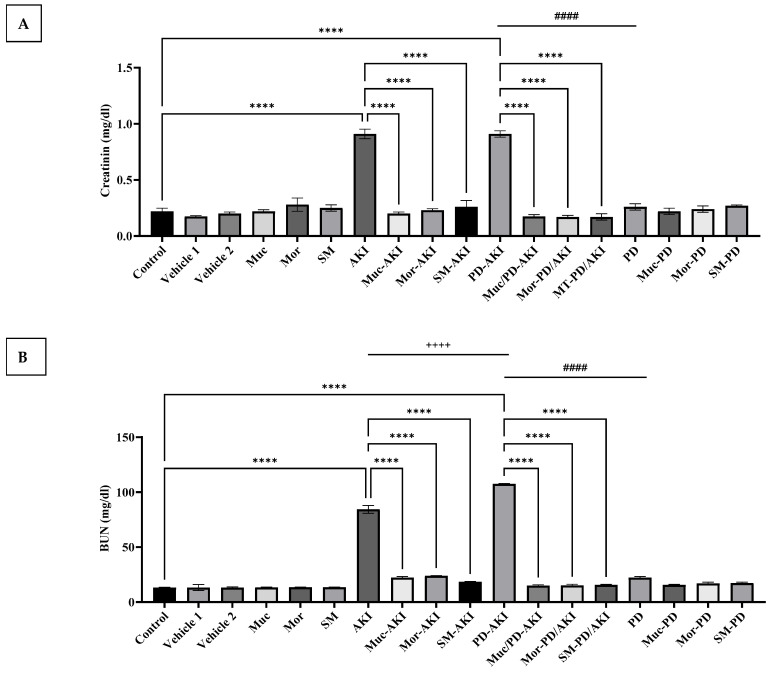
Effect of Muc, Mor, and SM extracts on renal function parameters. (**A**) shows serum creatinine levels (mg/dL) and (**B**) shows BUN levels (mg/dL) across all experimental groups. Data are presented as mean ± SEM (*n* = 3). (#) show comparison between PD-AKI vs. PD, and (+) show comparison between PD-AKI vs. AKI. **** *p* < 0.0001, #### *p* < 0.0001, and ++++ *p* < 0.0001.

Serum creatinine and BUN levels were evaluated to assess renal function across the different experimental groups, as shown in Figure 1A and Figure 1B, respectively.

In the control group, the creatinine level was within normal range (0.22 ± 0.02 mg/dL). Comparable values were observed in the vehicle 1 and 2, Muc, Mor, and SM groups, suggesting no renal impairment induced by the vehicles or any of the herbal pre-treatments, where the creatinine remained at baseline (0.17–0.28 mg/dL).

In the AKI model, the creatinine levels increased significantly by 4.1-fold (*p* < 0.0001) compared to the control group. Pre-treatment of the AKI mice with Muc, Mor, or SM significantly (*p* < 0.0001) attenuated this increase (*p* < 0.0001), reducing creatinine levels by approximately 70–80% compared to the untreated AKI group.

The combined PD–AKI model exhibited higher creatinine (0.91 mg/dL), representing a 4.13-fold (*p* < 0.0001) increase compared to the control group, and a 3.5-fold increase (*p* < 0.0001) compared to the PD group and the AKI group. Pre-treatment with the extracts inhibited the increase in the creatinine level and kept it at baseline.

On the other hand, PD induction did not induce changes in creatinine level compared to the control, nor co-treatment with the three extracts (Figure 1A). As for the BUN level (Figure 1B), it was within normal range in the control (13.23 ± 0.29 mg/dL). Its trend among the different groups was similar to that of creatinine. The levels in the control, vehicle 1 and 2, and the three extract groups were around 13 mg/dL. Induction of AKI elevated the BUN level to 100.4 mg/dL, representing a 7.5-fold increase compared to the control group. Extract pre-treatment markedly reduced BUN in the AKI model with Muc/Mor/SM–AKI, yielding 22, 24, and 18.4 mg/dL (*p* < 0.0001), representing a 4.5-, 4.2-, and 5.5-fold decrease, respectively, over the AKI group.

Similarly, PD-AKI induction triggered a significant elevation (107.5 mg/dL), representing an 8.1-fold (*p* < 0.0001) increase compared to the control group. When comparing the PD-AKI to the AKI group, a significant 1.3-fold change was noticed, and when comparing the PD-AKI to the PD group, a significant 4.8-fold change (*p* < 0.0001) was observed. Extract pre-treatment markedly reduced BUN in the Muc/Mor/SM–PD–AKI groups to 15, 15.2, and 15.6mg/dL (*p* < 0.0001), representing a 7.2-, 7-, and 6.9-fold decrease, respectively, compared to the PD-AKI group.

Again, PD induction did not induce any significant change in BUN levels nor treatment with the three extracts (Figure 1B) when compared to the control group. Based on the renal indices, AKI and PD–AKI both show marked kidney dysfunction, whereas PD alone remains near the control, indicating that rotenone does not drive primary renal failure without concomitant nephrotoxic insult.

### 2.2. Antioxidant Enzymes and Lipid Peroxidation in Kidney and Brain Tissues

**Figure 2:**
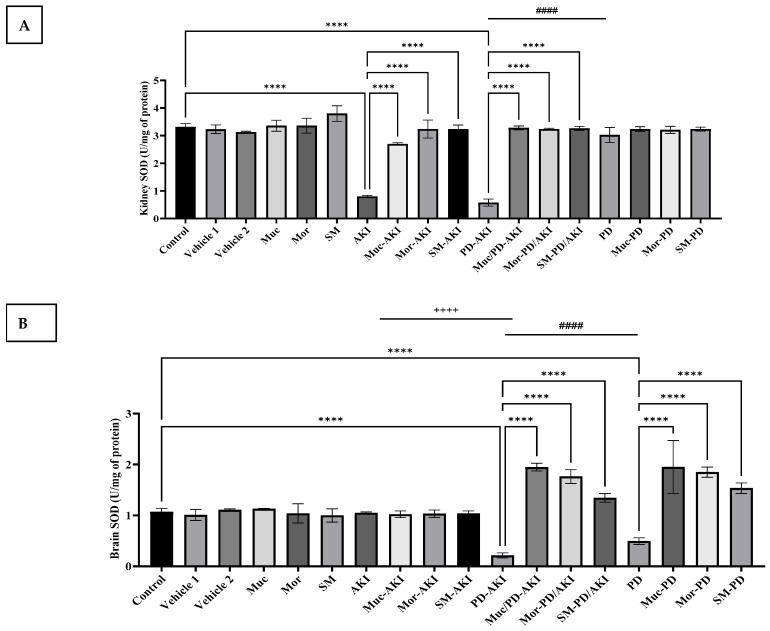
Effect of Muc, Mor, and SM extracts on SOD activity. SOD activity (U/mg of protein) in kidney tissue (**A**) and in brain tissue (**B**) across all experimental groups. Data are presented as mean ± SEM (*n* = 3). (#) show comparison between PD-AKI vs. PD, and (+) show comparison between PD-AKI vs. AKI. **** *p* < 0.0001, #### *p* < 0.0001, and ++++ *p* < 0.0001.

**Figure 3:**
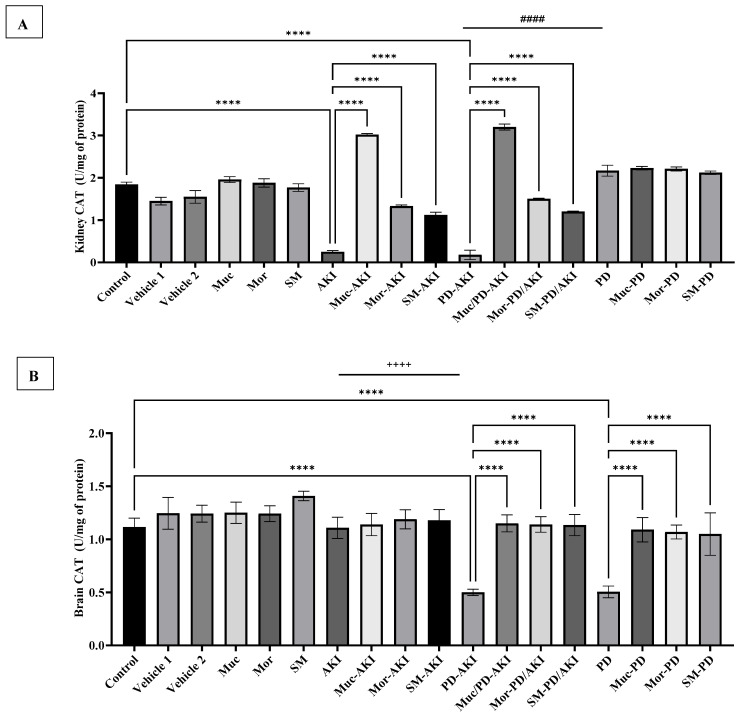
Effect of Muc, Mor, and SM extracts on CAT activity. CAT activity (U/mg of protein) in kidney tissue (**A**) and brain tissue (**B**) across all experimental groups. Data are presented as mean ± SEM (*n* = 3). (#) show comparison between PD-AKI vs. PD, and (+) show comparison between PD-AKI vs. AKI. **** *p* < 0.0001, #### *p* < 0.0001, and ++++ *p* < 0.0001.

**Figure 4:**
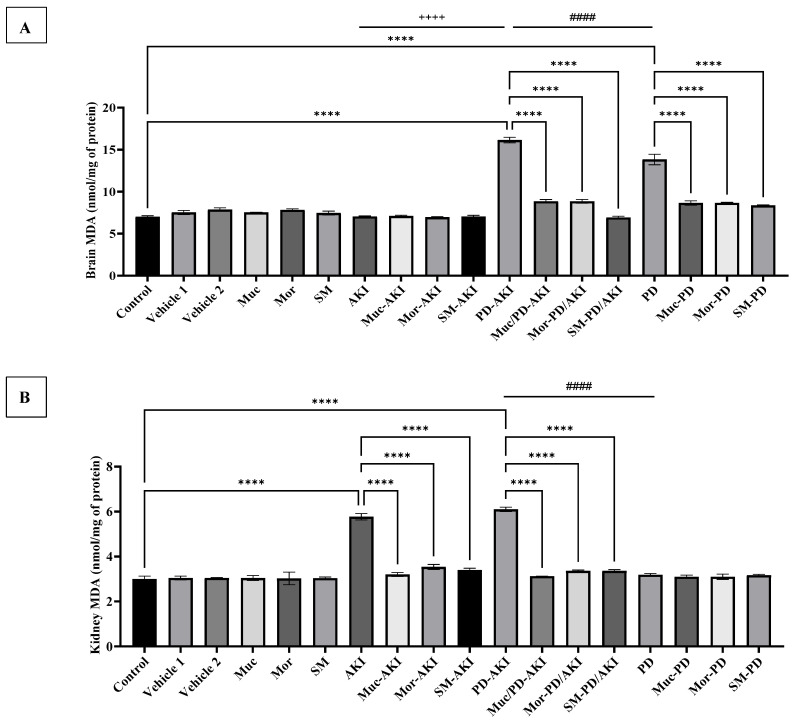
Effect of Muc, Mor, and SM extracts on MDA levels. MDA levels (nmol/mg of protein) in kidney tissue (**A**) and brain tissue (**B**) across all experimental groups. Data are presented as mean ± SEM (*n* = 3). (#) show comparison between PD-AKI vs. PD, and (+) show comparison between PD-AKI vs. AKI. **** *p* < 0.0001, #### *p* < 0.0001, and ++++ *p* < 0.0001.

To evaluate the effect of the three extracts on oxidative stress and antioxidant defense, the activities of SOD (Figure 2), CAT (Figure 3), and MDA (Figure 4) were measured in both kidney and brain tissues in the three disease models: AKI, PD-AKI, and PD.

#### 2.2.1. Superoxide Dismutase (SOD) Levels in Kidney and Brain Tissues

In the control group, in the kidneys, the SOD level was within the normal range (3.32 ± 0.11 mg/dL). Comparable values were observed in the vehicle 1 and 2, Muc, Mor, and SM groups, suggesting no renal impairment induced by the vehicles or any of the herbal pre-treatments, where the SOD levels remained at baseline (Figure 2A). AKI induction significantly decreased the levels of SOD in the kidneys by 4-fold (*p* < 0.0001) compared to the control group. However, pre-treating the AKI mice with all three extracts helped in maintaining elevated SOD levels, where a significant (*p* < 0.0001) increase was observed in SOD levels by 3.4-fold, 4-fold, and 4-fold with Muc, Mor, and SM treatments, respectively, compared to the untreated AKI group.

Similarly, in the PD-AKI model, a significant decrease in SOD levels by 5.7-fold (*p* < 0.0001) was observed compared to the control group. Pre-treating with the three extracts significantly restored the SOD level, restoring it to the normal baseline. Extract pre-treatment significantly increased SOD levels in the Muc/Mor/SM–PD–AKI groups by 5.7-, 5.6-, and 5.6-fold (*p* < 0.0001), respectively.

In the kidney, antioxidant collapse was evident in both the AKI and PD–AKI groups, without any statistical difference. While in the PD group, SOD levels were unchanged when compared to normal, it was significantly higher than those of the PD-AKI group by 5-fold (*p* < 0.0001). Also, no changes in SOD activity were observed, with the Muc/Mor/SM–PD groups aligning.

In the brain, SOD activity ranged between 1.07–1.14 U/mg protein in the control, vehicle 1, and vehicle 2 groups (Figure 2B), with no significant changes with the three extracts alone (Muc, Mor, and SM) (Figure 2B). Similarly, in the AKI model, SOD activity remained unaffected with or without extract treatment. However, in the PD model, a significant suppression of brain SOD by 2-fold was observed when compared to the control (*p* < 0.0001), confirming rotenone-induced oxidative burden. Co-treatment with the extract significantly (*p* < 0.0001) counteracted this decline by 3.9-, 3.7-, and 3-fold with Muc–PD, Mor–PD, and SM–PD, respectively, compared to the PD group.

In the dual PD–AKI model, SOD levels in the brain declined further by 4.8-fold (*p* < 0.0001) compared to the control group; yet, pre-treatment with the extracts substantially elevated SOD activities by 8.9-, 8-, and 6.13-fold in Muc–PD–AKI, Mor–PD–AKI, and SM–PD–AKI, respectively, nearly doubling the levels compared to the untreated PD–AKI (*p* < 0.0001). This large restoration underscores the strong antioxidant activities of three extracts.

In the brain, PD and PD–AKI both exhibited pronounced SOD activity, where it was significantly increased by 2-fold (*p* < 0.0001) compared to the PD group, with a 4.6-fold increase compared to the AKI group (*p* < 0.0001), showing synergistic oxidative damage when PD is compounded by AKI.

#### 2.2.2. Catalase (CAT) Activity in Kidney and Brain Tissues

Baseline renal CAT activity was between 1.8 ± 0.055 U/mg in the control group, and comparable values in the vehicle 1 (1.45 ± 0.099 U/mg) and vehicle 2 (1.55 ± 0.15 U/mg) groups (Figure 3A). The CAT activity in the groups treated with the extracts alone (Muc, Mor, and SM) showed no significant changes, indicating no deviation from the control (Figure 3A).

In the AKI model, renal CAT activity was significantly (*p* < 0.0001) suppressed (7.4-fold vs. the control, confirming the exhaustion of the antioxidative stress enzyme. Extract pre-treatment attenuated this reduction by 12-fold with Muc, 5.32-fold with Mor, and 4.48-fold with SM compared to the AKI group. In the PD–AKI model, CAT activity significantly dropped further (by 10-fold vs. the control, *p* < 0.0001), reflecting extensive oxidative stress. Notably, extract pre-treatment significantly (*p* < 0.0001) enhanced enzyme activity and minimized this reduction 17.7-fold with Muc, 8.33-fold with Mor, and 6.66-fold with SM. Collectively, these results confirm that the extracts preserve CAT activity under both AKI and PD–AKI stress conditions. When comparing the PD-AKI to the AKI group, a 2.5-fold increase (*p* < 0.0001) was observed, with a 12-fold increase (*p* < 0.0001) when compared to the PD group, thus reinforcing a kidney-centric oxidative burden when AKI and PD are present. In the PD group, renal CAT activity did not significantly change with or without extract treatment.

In the brain, the baseline CAT activity was 1.12 ± 0.085 U/mg protein. No significant changes were observed in the vehicle or extract-alone groups (Figure 3B).

In the AKI model, CAT levels remained near baseline with or without extract treatment, indicating minimal oxidative compromise in the brain, compared to the kidney.

In the PD–AKI model, CAT activity was markedly reduced by 2.3-fold (*p* < 0.0001). With the three extracts pre-treatment, it is significantly elevated by ~2.3-fold (*p* < 0.0001) compared to the untreated PD-AKI, showing marked recovery.

As for CAT activity in the PD group, a marked reduction by 2.1-fold vs. the control (*p* < 0.0001) was observed, confirming rotenone-induced depletion of antioxidant defense. Extract treatment significantly restored CAT activity in the Muc–PD, Mor–PD, and SM–PD groups by 2.1- to 2-fold, almost fully normalizing the activity. Brain CAT declined in PD and PD–AKI in parallel patterns, but not in AKI. AKI was significantly lower by 2.2-fold (*p* < 0.0001) when compared to PD-AKI, indicating that central antioxidant exhaustion tracks with the PD component rather than with isolated renal injury.

#### 2.2.3. Malondialdehyde Levels in Kidney and Brain Tissues

Renal malondialdehyde (MDA) levels (Figure 4A) in the control and vehicle groups were between 3.0 and 3.2 nmol/mg protein, representing baseline lipid peroxidation. The level of MDA in the extract groups was comparable to that of the control group (3.0–3.5 nmol/mg). However, AKI induction significantly increased MDA by 1.9-fold (*p* < 0.0001) compared to the control, consistent with oxidative lipid damage. Pre-treatment with Muc, Mor, or SM extracts significantly mitigated this effect, with values reduced by 1.8-, 1.6-, and 1.7-fold (*p* < 0.0001), respectively, nearly restoring control levels compared to the AKI group. The PD–AKI model showed a further rise in MDA by 2.0-fold (*p* < 0.0001) compared to the control group, surpassing AKI alone by a 0.33-fold change (*p* < 0.0001), and a 2.91-fold (*p* < 0.0001) increase compared to the PD group. However, extract treatment again was efficient in minimizing oxidative stress, where the Muc–PD–AKI, Mor–PD–AKI, and SM–PD–AKI groups displayed lower MDA levels of ~1.8–1.9-fold reduction compared to the untreated PD–AKI (*p* < 0.0001). These findings confirm that the extracts efficiently prevented lipid peroxidation across both single and dual injury models. In the PD group, renal MDA activity sustained values close to the control group; a similar pattern was observed with the three-extract co-treatment, which maintained values comparable to the control group.

In the brain, the baseline MDA level was 7.02 ± 0.11 nmol/mg of protein without any significant change between the control, vehicle, and extracts groups (Figure 4B). AKI induction did not substantially alter MDA levels, consistent with minimal oxidative burden on the brain under kidney-only stress. The levels of MDA in the extract pre-treated AKI groups also remained at near-basal levels (7.04–7.46 nmol/mg). The PD–AKI model showed a great increase in MDA level by 2.3-fold compared to the control (*p* < 0.0001), suggesting intensified oxidative damage when both injuries coexist. Again, extract pre-treatment produced significant (*p* < 0.0001) protective effects with a 1.8–2.3-fold decrease in MDA levels in Muc–PD–AKI, Mor–PD–AKI, and SM–PD–AKI, restoring levels to control values. These findings confirm that the three extracts are potent inhibitors of lipid peroxidation in both isolated PD and comorbid PD–AKI conditions. Furthermore, the PD model also induced a significant increase in MDA by 1.97-fold compared to the control group (*p* < 0.0001), indicating severe lipid peroxidation. Co-treatment with the extracts significantly (*p* < 0.0001) attenuated this elevation in the Muc–PD, Mor–PD, and SM–PD groups by 1.6-fold, compared to the untreated PD group. Brain MDA peaks in PD-AKI when compared to PD (*p* < 0.0001) by 1.2-fold, while AKI alone is near baseline and significantly lower than PD-AKI by 2.3-fold, highlighting synergistic central lipid damage when PD is compounded by AKI.

### 2.3. Inflammatory Cytokines in Kidney and Brain Tissues

**Figure 5:**
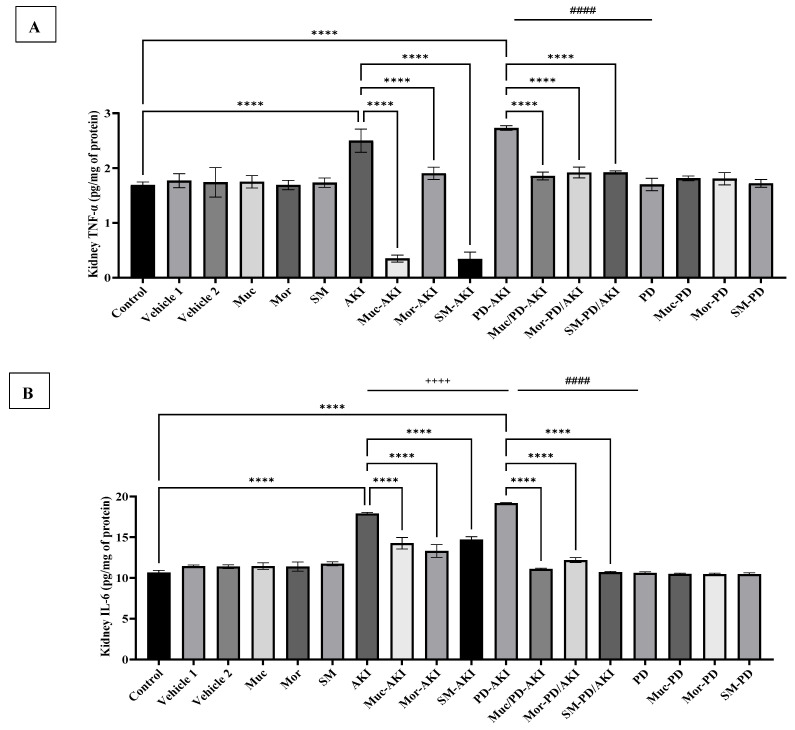
Effect of Muc, Mor, and SM extracts on IL-6 and TNF-α levels in kidney tissue. (**A**) shows the IL-6 levels (pg/mg of protein) and (**B**) shows the TNF-α levels (pg/mg of protein) across all experimental groups. Data are presented as mean ± SEM (*n* = 3). (#) show comparison between PD-AKI vs. PD, and (+) show comparison between PD-AKI vs. AKI. **** *p* < 0.0001, #### *p* < 0.0001, and ++++ *p* < 0.0001.

To assess the effect of the three extracts on inflammation in both the kidney and brain tissues, the levels of IL-6 and TNF-α were evaluated. Figure 5A shows the impact of the prophylactic administration of the extracts on renal IL-6 concentration. The baseline renal IL-6 level was 10.67 ± 0.25 pg/mg protein in the control group, similar to that of both vehicles 1 and 2, which ranged between 11.3 and 11.5 pg/mg. Administration of the Muc, Mor, or SM extracts to normal mice did not significantly change IL-6 levels, confirming that the extracts do not alter basal levels of this cytokine. In contrast, AKI induction triggered a significant increase in this pro-inflammatory marker, surging to 1.67-fold compared to the control group (*p* < 0.0001). Pre-treatment with the extracts markedly attenuated this elevation in the Muc–AKI, Mor–AKI, and SM–AKI groups by a 1.25-, 1.3-, and 1.2-fold decline (*p* < 0.0001) compared to the AKI group. The dual PD–AKI model provoked the most pronounced response, with IL-6 reaching 19.2 pg/mg, a 1.8-fold increase compared to the control (*p* < 0.0001). Pre-treatment with the extracts demonstrated strong anti-inflammatory efficacy, effectively normalizing the inflammatory response to near-control values, where the levels of this cytokine were reduced around 1.6- to 1.7-fold in the Muc–PD–AKI, Mor–PD–AKI, and SM–PD–AKI groups compared to the PD-AKI group. As for the PD model, it did not produce an inflammatory burst, with IL-6 remaining within the baseline range, and the extract co-treatment in the PD groups (Muc–PD, Mor–PD, and SM–PD) maintained similarly low levels, underscoring their stability under rotenone challenge in the kidney. AKI and PD–AKI elicited robust renal IL-6 responses, whereas PD alone did not induce a change, confirming that kidney inflammation is injury-led rather than rotenone-led. PD–AKI surpasses AKI in cytokine magnitude by 1.07-fold (*p* < 0.0001), and PD by 1.8-fold (*p* < 0.0001), consistent with systemic inflammatory amplification when both organs are compromised.

Figure 5B shows the impact of Muc, Mor, and SM pre-treatment on renal TNF-α levels in the three disease models: AKI, PD-AKI, and PD. The baseline of renal TNF-α levels was 1.69 ± 0.06 pg/mg protein in the control group, as in both vehicle groups 1 and 2, which ranged between 1.69 and 1.77 pg/mg. In the AKI model, the level of TNF-α in the AKI group increased by 1.5-fold compared to the control group, confirming renal inflammation following AKI induction. Pre-treatment with Muc, Mor, and SM reduced TNF-α by 7.14-, 1.3-, and 7.4-fold, respectively, compared to the AKI group. In the PD-AKI model, TNF-α significantly reached 1.6-fold higher than the control group, and 1.6-fold higher than PD (*p* < 0.0001), indicating intensified inflammation from the dual-organ insult. Both AKI and PD-AKI showed similar patterns. Muc/PD-AKI reduced TNF-α by 1.5-fold, while Mor/PD-AKI and SM/PD-AKI lowered it each by 1.4-fold compared to the untreated PD-AKI group. In the PD model, no significant changes were observed with and without the extract treatment compared to the control group.

**Figure 6:**
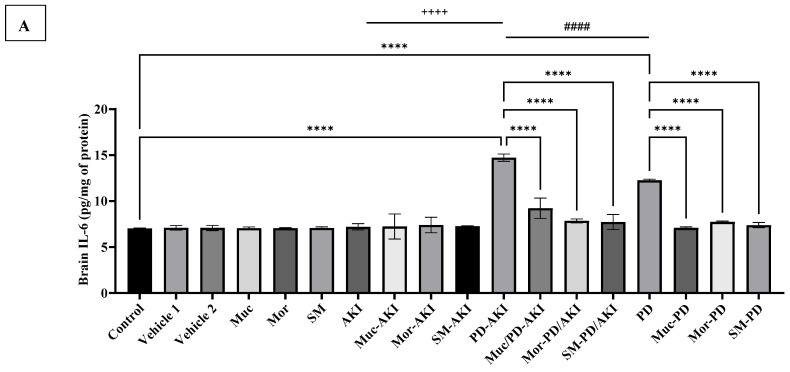

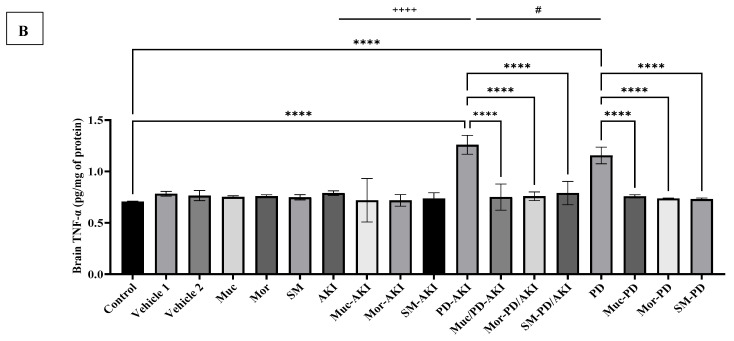
Effect of Muc, Mor, and SM extracts on IL-6 levels and TNF-α levels in brain tissue. (**A**) shows the IL-6 levels (pg/mg of protein) and (**B**) shows the TNF-α levels (pg/mg of protein) across all experimental groups. Data are presented as mean ± SEM (*n* = 3). (#) show comparison between PD-AKI vs. PD, and (+) show comparison between PD-AKI vs. AKI. **** *p* < 0.0001, # *p* < 0.05, #### *p* < 0.0001, and ++++ *p* < 0.0001.

At the brain level, Figure 6A shows the effect of the three extracts on IL-6 concentrations across the three disease models: AKI, PD-AKI, and PD. In the control group, the baseline brain IL-6 levels were 7.0 pg/mg protein, and this was maintained across both vehicle groups (7.08–7.10 pg/mg). Administration of Muc, Mor, or SM did not alter IL-6, confirming that the extracts exerted no pro-inflammatory effect under physiological conditions. In the AKI model, IL-6 levels were comparable to those of the control, with or without the extracts. In contrast, the dual PD–AKI model showed the most severe inflammatory response, with IL-6 reaching 14.7 pg/mg, a 2.1-fold increase compared to the control (*p* < 0.0001). Again, all three extracts demonstrated significant efficacy, where in the Muc–PD–AKI, Mor–PD–AKI, and SM–PD–AKI groups, IL-6 levels were reduced to 7.7–9.2 pg/mg protein, a 1.6- to 2.0-fold decline compared to the untreated PD–AKI. Similarly, the PD model induced a pronounced elevation of brain IL-6 by 1.7-fold compared to the control (*p* < 0.0001), consistent with rotenone-induced neuroinflammation. The extract co-treatment strongly suppressed this surge, reducing IL-6 by 1.6 to 1.7-fold in the Muc–PD, Mor–PD, and SM–PD groups compared to PD alone. Brain IL-6 increased in PD-AKI by 1.2-fold when compared to PD (*p* < 0.0001), and 2.2-fold (*p* < 0.0001) when compared to AKI, highlighting synergistic brain inflammation when PD is compounded by AKI.

The brain TNF-α level of the control group was 0.71 pg/mg protein, as shown in Figure 6B. Both vehicle 1 and 2 groups showed comparable values (0.77–0.78 pg/mg protein). Administration of Muc, Mor, or SM did not produce any significant changes in TNF-α levels, confirming that their use does not induce any pro-inflammatory activity under normal conditions. In the AKI model, brain TNF-α remained unaltered, suggesting that renal injury alone did not strongly trigger central TNF-α elevation. Similarly, the AKI groups treated with the extracts (Muc–AKI, Mor–AKI, and SM–AKI) maintained TNF-α levels close to the control (0.72–0.79 pg/mg), reflecting the extracts’ stabilizing role. The dual PD–AKI model provoked a significant rise in TNF-α levels, a 1.8-fold rise compared to the control (*p* < 0.0001). The level of TNF-α in PD–AKI was significantly higher, by 1.08-fold (*p* < 0.05) when compared to PD, and by 1.59-fold when compared to the AKI group, which was close to the control group, indicating that the brain inflammatory signal is PD-dependent. However, the extract pre-treatment significantly countered this effect. The Muc–PD–AKI, Mor–PD–AKI, and SM–PD–AKI groups yielded values of 0.75–0.79 pg/mg (~1.6-fold reduction; *p* < 0.0001) compared to the untreated PD–AKI. Similarly, the PD model induced a marked increase in TNF-α by 1.6-fold compared to the control (*p* < 0.0001), indicating rotenone-driven neuroinflammation. Co-treatment with the extracts effectively suppressed this rise, where TNF-α levels in the Muc–PD, Mor–PD, and SM–PD groups ranged between 0.73 and 0.76 pg/mg protein (~1.5-fold decrease), fully restoring cytokine levels toward baseline. These findings confirm that while AKI alone does not induce inflammatory responses in the brain, PD and PD–AKI induction provoke robust ones. Importantly, the Muc, Mor, and SM extracts demonstrated powerful anti-inflammatory efficacy, restoring TNF-α to near-control levels under both single and combined injury conditions.

### 2.4. Gene Expression Profiling

The effect of the three extracts on gene expression was also studied in the three experimental models.

#### 2.4.1. KIM-1 Gene Expression

**Figure 7:**
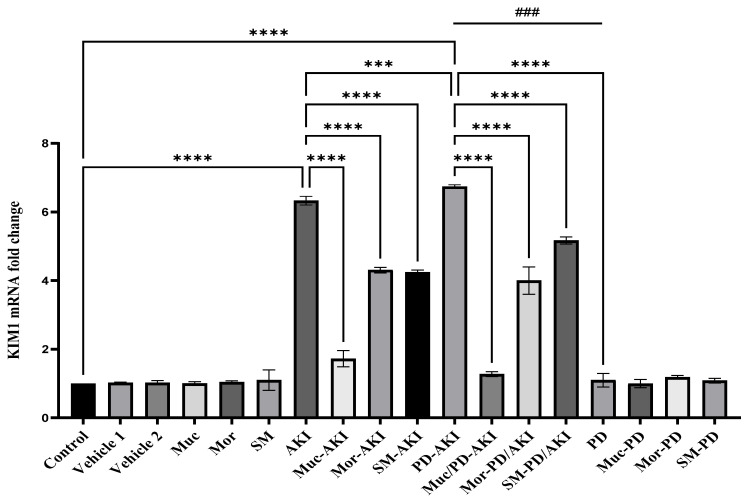
Effect of Muc, Mor, and SM extracts on *KIM1* expression. Data are presented as mean ± SEM (*n* = 3). (#) show comparison between PD-AKI vs. PD vs. AKI. *** *p* < 0.001, **** *p* < 0.0001, and ### *p* < 0.001.

The expression of *kidney injury molecule-1 (KIM-1)* in all the experimental groups is shown in Figure 7. In the control, vehicle, and extracts groups, *KIM-1* expression remained at a basal level, with a fold change ranging between 1 and 1.1, confirming that the extracts did not cause any renal injury under physiological conditions. In the AKI model, *KIM-1* was significantly overexpressed at 6.3-fold compared to the control (*p* < 0.0001), validating the onset of acute renal damage. Pre-treatment with the extracts markedly mitigated this response, where the Muc–AKI, Mor–AKI, and SM–AKI groups exhibited reductions between 1.5- and 3.6-fold compared to the untreated AKI. Similarly, the dual PD–AKI model caused a strong induction of *KIM-1* expression, with a 6.7-fold increase compared to the control (*p* < 0.0001). The PD–AKI rise is comparable to AKI, and more than PD by 6.0-fold (*p* < 0.001), pinpointing AKI-driven tubular injury. The extract pre-treatment robustly countered this effect, reducing *KIM-1* expression levels in Muc–PD–AKI, Mor–PD–AKI, and SM–PD–AKI to 1.3–5.3-fold, compared to the untreated PD–AKI. The PD model alone did not significantly alter *KIM-1* expression (1.10-fold vs. the control), and co-treating the PD mice with the extracts (Muc–PD, Mor–PD, and SM–PD) maintained near-baseline expression (1.0–1.2 fold).

#### 2.4.2. Nrf2 Gene Expression

**Figure 8:**
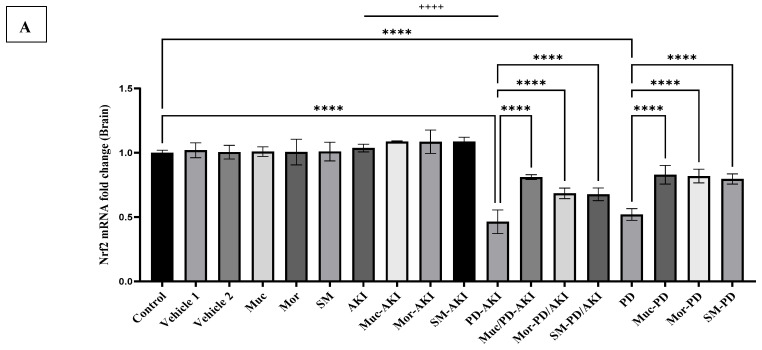

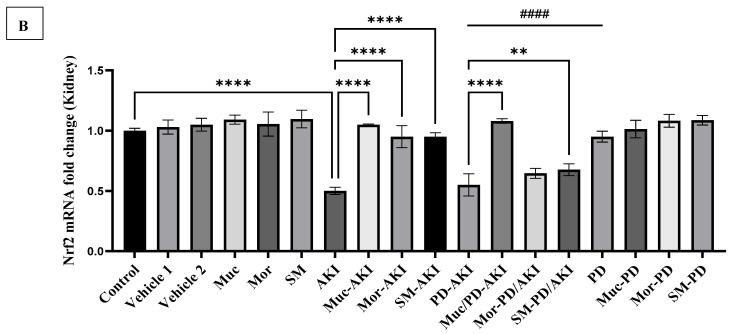
The effect of the Muc, Mor, and SM extracts on *Nrf2* expression in the kidney (**A**) and brain (**B**). Data are presented as the mean ± SEM (*n* = 3). (#) show the comparison between PD-AKI vs. PD, and (+) show the comparison between PD-AKI vs. AKI. ** *p* < 0.01, **** *p* < 0.0001, ++++ *p* < 0.0001, and #### *p* < 0.0001.

The mRNA expression levels of *nuclear factor erythroid 2-related factor 2 (Nrf2)* were evaluated across all experimental groups to assess the oxidative stress response in kidney tissue (Figure 8A) and brain tissue (Figure 8B).

In kidney tissue, in the control and vehicle groups, renal *Nrf2* expression remained steady at baseline (Figure 8A). Administration of Muc, Mor, or SM alone did not alter this expression, suggesting no extract-related perturbation under physiological conditions. In the AKI model, *Nrf2* expression was significantly downregulated by 2.0-fold (*p* < 0.0001) compared to the control group, implying suppression of antioxidant defense pathways under acute renal stress. Pre-treatment with the extracts significantly countered this decline, where the Muc–AKI, Mor–AKI, and SM–AKI groups displayed restored levels of 1.9–2.1-fold (*p* < 0.0001) compared to the AKI group, effectively normalizing *Nrf2* activity. The dual PD–AKI model exerted an inhibitory effect, with *Nrf2* expression reduced by 1.8-fold compared to the control (*p* < 0.0001). Renal Nrf2 was similarly depressed in both AKI and PD–AKI, without any difference, but was significantly lower by 1.8-fold compared to the PD group. The extract pre-treatment in these animals provided substantial rescue. Muc–PD–AKI, Mor–PD–AKI, and SM–PD–AKI increased expression to 1.9-, 1.2-, and 1.2-fold compared to the untreated PD-AKI group, respectively, reflecting nearly complete normalization with Muc, and partial but significant improvement with Mor and SM. In the PD model, no significant changes were observed, neither in the PD group nor the treated groups.

In the brain, no significant differences were observed among the control, vehicle, extracts (Muc, Mor, and SM) groups, as well as the AKI model group with or without treatment (Figure 8B). However, in the PD-AKI model, the PD-AKI group demonstrated a notable reduction in *Nrf2* expression (2.1-fold, *p* < 0.0001), indicating a significant impairment in the endogenous antioxidant defense system under the combined stress of PD and AKI. Both PD and PD–AKI downregulate *Nrf2* in a similar trend, while in the AKI group, levels remained close to the control, and significantly higher by 2.2-fold (*p* < 0.0001) when compared to PD-AKI. The downregulation reinforces the combined neuroinflammatory and oxidative stress resulting from dual pathology, which likely surpasses *Nrf2*-mediated cytoprotective mechanisms. Treatment with Muc, Mor, and SM in the PD-AKI mice partially reversed the observed suppression, with Muc/PD-AKI showing the most significant improvement (1.7-fold, *p* < 0.0001), followed by Mor/PD-AKI (1.5-fold, *p* < 0.0001) and SM/PD-AKI (1.5-fold, *p* < 0.0001), compared to the untreated PD-AKI group. In the PD model, the PD group exhibited significant *Nrf2* suppression (1.9-fold, *p* < 0.0001), indicating disrupted redox homeostasis in the affected brain. The three extracts markedly reduced this decline, with the Muc and Mor extract exhibiting the highest neuroprotective capacity (1.6-fold, *p* < 0.0001) and SM-PD (1.5-fold, *p* < 0.0001), indicating a consistent pattern of *Nrf2* modulation in both PD and PD-AKI contexts.

#### 2.4.3. NF-κB Gene Expression

**Figure 9:**
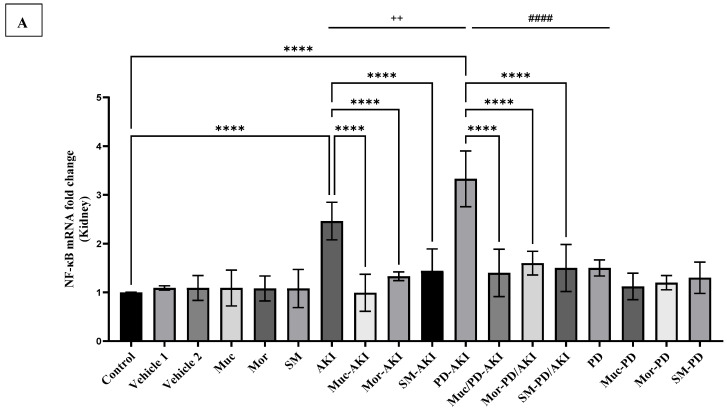

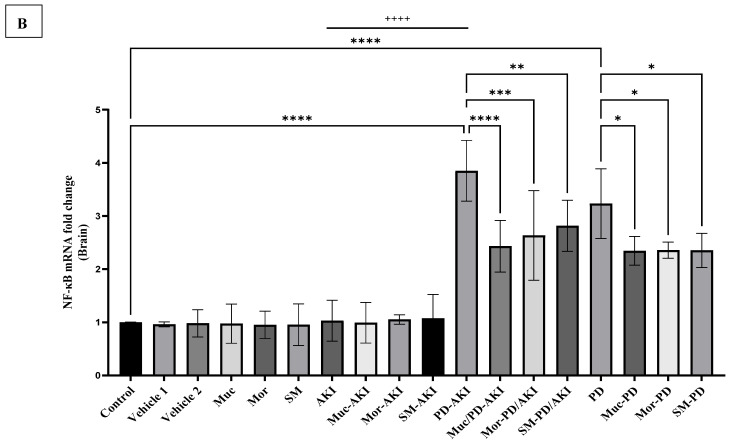
The effect of the Muc, Mor, and SM extracts on *NF-κB* mRNA expression in the kidney (**A**) and brain (**B**). Data are presented as the mean ± SEM (*n* = 3). (#) show the comparison between PD-AKI vs. PD, and (+) show the comparison between PD-AKI vs. AKI. * *p* < 0.05, ** *p* < 0.01, *** *p* < 0.001, **** *p* < 0.0001, ++ *p* < 0.01, ++++ *p* < 0.0001, and #### *p* < 0.0001.

The mRNA levels of *nuclear factor kappa B (NF-κB)* were evaluated to examine the inflammatory response in kidney tissue (Figure 9A) and brain tissue (Figure 9B). In the control and vehicle groups, *NF-κB* mRNA expression remained basal (1.0–1.09-fold). Administration of the three extracts did not significantly alter expression. In the AKI model, *NF-κB* expression was markedly upregulated (2.5-fold vs. control, *p* < 0.0001), reflecting strong inflammatory activation in renal tissue. The extract pre-treatment substantially suppressed this elevation. In the Muc–AKI group, a significant reduction in the expression levels by 2.5-fold was observed compared to the AKI group, while in the Mor–AKI group, the change was a 1.84-fold decrease, and in the SM–AKI group, a 1.7-fold decrease was observed compared to the AKI group. The Mor and SM extracts led to partial reduction, indicating protective but somewhat less potent regulation compared to the Muc extract in treating AKI. The dual PD–AKI model induced the strongest inflammatory activation, with *NF-κB* expression levels surging by 3.3-fold (*p* < 0.0001). The renal *NF-κB* activation was more prominent in PD–AKI than AKI by 1.4-fold (*p* < 0.05), and by 2.2-fold than PD, matching the renal cytokine profile and locating inflammation to the AKI axis. However, the extract pre-treatment, again, produced marked attenuation. Muc–PD–AKI, Mor–PD–AKI, and SM–PD–AKI reduced expression by 2.4-, 2.1-, and 2.2-fold, respectively, when compared to the untreated PD–AKI. In the PD model, no significant changes in *NF-κB* expression were observed in the untreated or in the treated groups.

In brain tissue (Figure 9B), *NF-κB* was significantly upregulated in both the PD (3.2-fold, *p* < 0.0001) and PD-AKI groups (3.8-fold, *p* < 0.0001) with respect to the control. Pre-treatment with the Muc, Mor, and SM extracts reduced *NF-κB* overexpression in both the PD and PD-AKI models, though the extent of reduction varied. Muc/PD-AKI demonstrated significant suppression (1.6-fold, *p* < 0.0001) compared to the untreated PD-AKI group. Mor/PD-AKI and SM/PD-AKI significantly reduced *NF-κB* levels by 1.5-fold (*p* < 0.0001) and 1.4-fold (*p* < 0.0001), respectively. However, none of the treatments completely restored basal expression levels. In the PD model, the Muc, Mor, and SM co-treatments reduced *NF-κB* levels by 1.4-fold (*p* < 0.05) when compared to the PD group, indicating a partial reversal of inflammation, with the Muc extract demonstrating superior efficacy compared to the others. The sustained elevation indicated ongoing neuroinflammatory activity that phytotherapy has not fully attenuated, highlighting the intricate and enduring characteristics of inflammation associated with PD. Both PD and PD–AKI showed strong NF-κB upregulation, with PD–AKI being the highest (*p* < 0.0001), whereas AKI showed comparable values to the control group, and less by 3.7-fold if compared to PD-AKI, indicating PD-driven central inflammatory signaling that is amplified by AKI.

#### 2.4.4. PI3K Gene Expression

**Figure 10:**
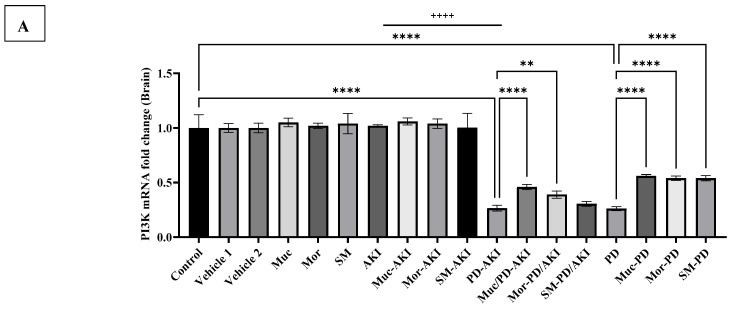

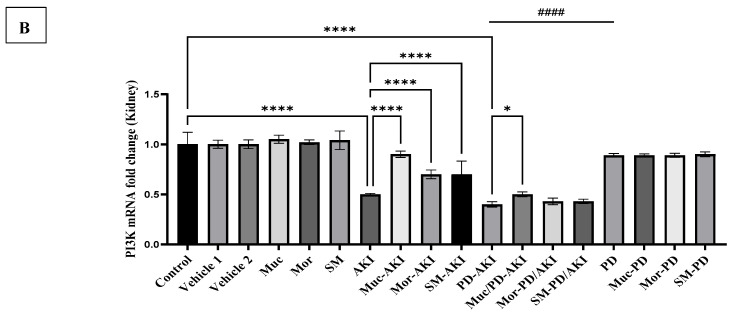
The effect of the Muc, Mor, and SM extracts on *PI3K* mRNA expression in the kidney (**A**) and brain (**B**). Data are presented as the mean ± SEM (n = 3). (#) show the comparison between PD-AKI vs. PD, and (+) show the comparison between PD-AKI vs. AKI.* *p* < 0.05, ** *p* < 0.01, **** *p* < 0.0001, ++++ *p* < 0.0001, and #### *p* < 0.0001.

The expression of *phosphoinositide 3-kinase (PI3K)* was quantified across all experimental groups to assess the status of cell survival signaling in kidney tissue (Figure 10A) and brain tissue (Figure 10B).

In kidney tissue, in the control and vehicle groups, *PI3K* expression was stable at baseline. The extract-alone groups (Muc, Mor, and SM) similarly showed no changes (1.02–1.05-fold), confirming no basal modulation. In the AKI model, *PI3K* expression was significantly downregulated by 2.0-fold (*p* < 0.0001) compared to the control group, consistent with impaired survival signaling in renal tissue. The extract pre-treatment improved this suppression: Muc–AKI restored expression to 1.8-fold, while Mor–AKI and SM-AKI to 1.4-fold. The PD model showed no significant changes. The dual PD–AKI model caused severe suppression of *PI3K* (2.5-fold, *p* < 0.0001). In the Muc–PD–AKI, Mor–PD–AKI, and SM–PD–AKI groups, expression was restored to 1.2-, 1.4-, and 1.4-fold, respectively, compared to the untreated PD–AKI. Renal *PI3K* was suppressed similarly in AKI and PD–AKI (PD-AKI by 1.3-fold more, if compared to AKI), but not in PD (2.2-fold difference when compared to PD-AKI), consistent with AKI-linked survival pathway impairment in the kidney.

In the brain, the levels of *PI3K* mRNA were consistent across the control, vehicle, and AKI groups, as well as in single-treatment and pre-treatment conditions (Muc, Mor, and SM, and their combinations with AKI) (Figure 10B). In sharp contrast, the PD model provoked a significant downregulation of *PI3K* (3.8-fold vs. control, *p* < 0.0001), indicating a strong suppression of neuronal survival signaling. Co-treatment with the extracts partially minimized this decline. The Muc–PD, Mor–PD, and SM–PD animals enhanced the *PI3K* expression values by 2.1-fold compared to the untreated PD. The dual PD–AKI model produced an even more severe inhibition, with *PI3K* reduced by 3.7-fold (*p* < 0.0001). Brain *PI3K* decreased similarly in PD and PD–AKI, but not in AKI (less by a 3.9-fold difference when compared to PD-AKI), aligning survival–signaling loss with PD pathology, potentiated by dual stress. Once again, the extract pre-treatment induced significant recovery. The restoration of expression, ranging from 1.1-fold (*p* < 0.0001) to 1.7-fold (*p* < 0.01), was observed in the Muc–PD–AKI and Mor–PD–AKI groups, respectively, when compared to the untreated PD–AKI.

#### 2.4.5. AKT Gene Expression

**Figure 11:**
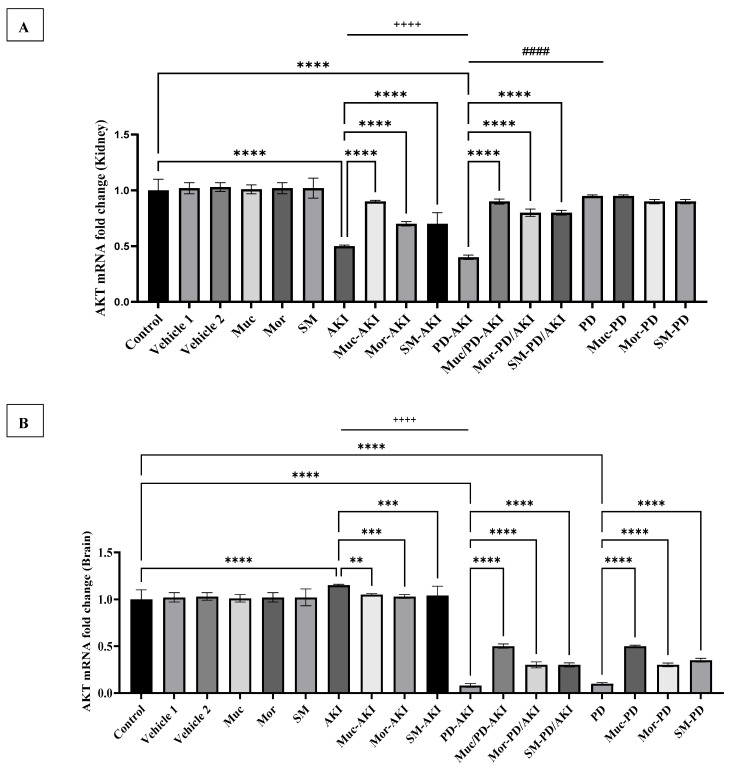
The effect of the Muc, Mor, and SM extracts on *AKT* mRNA expression in the kidney (**A**) and brain (**B**). Data are presented as the mean ± SEM (*n* = 3). (#) show the comparison between PD-AKI vs. PD, and (+) show the comparison between PD-AKI vs. AKI. ** *p* < 0.01, *** *p* < 0.001, **** *p* < 0.0001, ++++ *p* < 0.0001, and #### *p* < 0.0001.

To further investigate the downstream signaling of the PI3K pathway, the expression of *AKT*, a key kinase in the cell survival pathway, was assessed and is presented in Figure 11A for kidney tissue and Figure 11B for brain tissue. In the kidney, in the control and vehicle groups, *AKT* mRNA expression was stable, and the extract-alone groups (Muc, Mor, and SM) maintained similar values, confirming no basal modulation of *AKT* under physiological conditions. In the AKI model, *AKT* was markedly downregulated by 2.0-fold (*p* < 0.0001), consistent with impaired pro-survival signaling during acute renal injury. The pre-treatment with the extracts substantially attenuated this suppression. Muc–AKI restored *AKT* expression to 1.8-fold, while Mor–AKI and SM–AKI groups partially improved it to 1.4-fold. These results indicate that all three extracts mitigated AKI-induced *AKT* inhibition, with Muc showing stronger effects.

In the PD model, in the PD group and the extract co-treatment (Muc–PD, Mor–PD, and SM–PD), *AKT* expression remained close to baseline without any significant changes. By contrast, the dual PD–AKI model triggered the most pronounced suppression, reducing *AKT* expression by 2.5-fold vs. the control (*p* < 0.0001). Notably, the extract pre-treatment significantly rescued expressions: Muc–PD–AKI restored *AKT* to 2.5-fold, while Mor–PD–AKI and SM–PD–AKI improved it to 2.2-fold and 2.0-fold, respectively. Renal *AKT* downregulation was observed in AKI and PD–AKI, but not in PD. Whereas, in PD-AKI, it was increased by 1.2-fold (*p* < 0.0001) when compared to AKI and by 2.4-fold (*p* < 0.0001) when compared to PD, mirroring the renal *PI3K* pattern.

In the brain, *AKT* expression (Figure 11B) in the control, vehicle, and extract-alone groups (Muc, Mor, and SM) showed no alterations in expression. In the AKI model, AKT expression remained near baseline, suggesting that acute renal stress does not affect central *AKT* signaling. Pre-treatment with the extracts (Muc–AKI, Mor–AKI, and SM–AKI) sustained similar values (1.03–1.05-fold). In contrast, the PD model caused profound downregulation of *AKT* expression by 10-fold vs. the control (*p* < 0.0001), highlighting rotenone-induced impairment of neuronal survival pathways. Co-treatment with the extracts markedly alleviated this suppression. The Muc–PD, Mor–PD, and SM–PD groups restored expression to 5.0-, 3.0-, and 3.5-fold, respectively, compared to the untreated PD. The dual PD–AKI model produced the most dramatic suppression, with *AKT* reduced by 12.5-fold (*p* < 0.0001). Brain *AKT* was markedly and similarly reduced in the PD–AK and PD groups. When PD-AKI was compared to AKI, a significant 12.4-fold (*p* < 0.0001) decrease was observed, reinforcing a PD-driven neuronal survival deficit exacerbated by AKI. The extract pre-treatment provided significant recovery. Muc–PD–AKI, Mor–PD–AKI, and SM–PD–AKI restored expression to 6.2-, 3.7-, and 3.7-fold, respectively, compared to the untreated PD–AKI mice.

#### 2.4.6. mTOR Gene Expression

**Figure 12:**
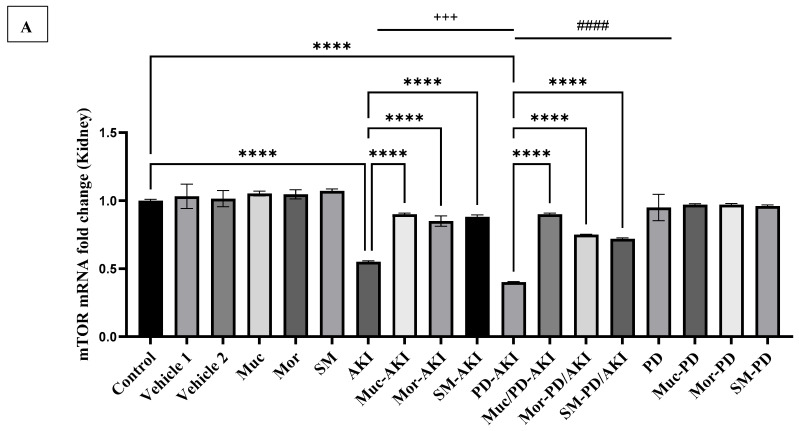

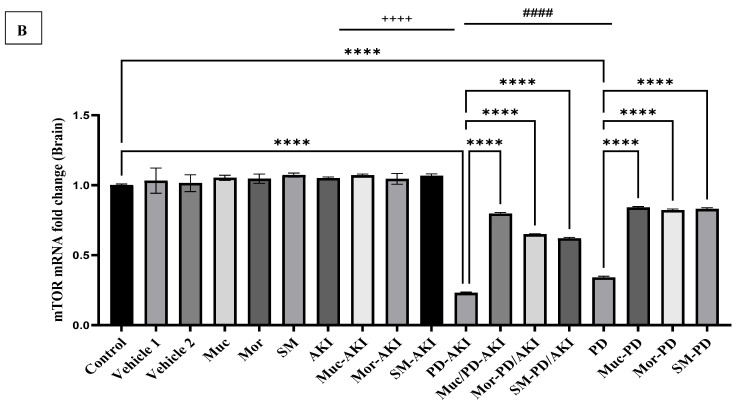
The effect of the Muc, Mor, and SM extracts on *mTOR* mRNA expression in the kidney (**A**) and brain (**B**). Data are presented as the mean ± SEM (n = 3). (#) show the comparison between PD-AKI vs. PD, and (+) show the comparison between PD-AKI vs. AKI. **** *p* < 0.0001, +++ *p* < 0.001, ++++ *p* < 0.0001, and #### *p* < 0.0001.

To finalize the evaluation of the PI3K/AKT/mTOR signaling pathway, the expression levels of the *mammalian target of rapamycin (mTOR)* were analyzed in the kidney (Figure 12A) and brain (Figure 12B).

In the control and vehicle groups, kidney *mTOR* expression remained steady at baseline (1.0–1.0-fold) (Figure 12A). The extract-alone groups (Muc, Mor, and SM) similarly showed no significant deviation (1.0-fold). In the AKI model, *mTOR* expression was markedly reduced by 1.8-fold (*p* < 0.0001), reflecting the suppression of survival and growth-related signaling in renal tissue. Pre-treatment with the extracts provided significant recovery. The expression levels in the Muc–AKI, Mor–AKI, and SM–AKI groups were restored to 1.6-, 1.5-, and 1.6-fold, respectively. The PD model alone showed no significant changes, the same as the co-treated extract groups. In sharp contrast, the dual PD–AKI model exhibited suppression of *mTOR*, reaching 2.5-fold vs. the control (*p* < 0.0001). Renal *mTOR* suppression was also observed in AKI and was higher by 1.4-fold in PD–AKI (*p* < 0.0001). In the PD group, the levels remained comparable to the control group, and when compared to PD-AKI, a significant 2.2-fold (*p* < 0.0001) increase was observed, paralleling the PI3K/AKT renal trends. The extract pre-treatment substantially alleviated this downregulation, where restoration of expression was observed in Muc–PD–AKI, Mor–PD–AKI, and SM–PD–AKI to 2.2-, 1.8-, and 1.8-fold, respectively, compared to the untreated PD–AKI.

In the brain tissue, *mTOR* mRNA levels were comparable across the control, vehicle, AKI, and treatment-only groups (Muc, Mor, and SM), as well as in their combinations with AKI, as shown in Figure 12B, suggesting stable homeostatic signaling via the PI3K/AKT/mTOR axis in the absence of neurodegenerative stress. A significant reduction in *mTOR* expression was noted in the PD-AKI group (4.3-fold, *p* < 0.0001), followed by the PD-only group (2.9-fold, *p* < 0.0001), compared to the control group. Pre-treatment with the three extracts demonstrated significant protective effects. In the PD-AKI groups, Muc/PD-AKI increased the *mTOR* expression by 3.4-fold (*p* < 0.0001) compared to the PD-AKI group, significantly reversing the transcriptional downregulation. Mor/PD-AKI and SM/PD-AKI demonstrated moderate recovery rates of 2.8-fold and 2.7-fold, respectively, compared to the PD-AKI group (*p* < 0.0001), yet they were less effective than the Muc extract pre-treatment. In the PD model groups, *mTOR* levels increased to 2.47, 2.41, and 2.44 (*p* < 0.0001) after treatment with the Muc, Mor, and SM extracts, respectively, showing significant recovery. Brain *mTOR* declined in PD and further in PD–AKI (1.5-fold, *p* < 0.0001), with the levels in the AKI group close to the control, indicating PD-linked impairment of neuronal growth/autophagy signaling intensified by dual pathology.

### 2.5. Behavioral Assessment

Behavioral assessments are essential for evaluating motor deficits in experimental models of PD. Among these, the rotarod and pole tests are widely used to assess motor coordination, balance, and bradykinesia in rodents.

#### 2.5.1. Rotarod

**Figure 13:**
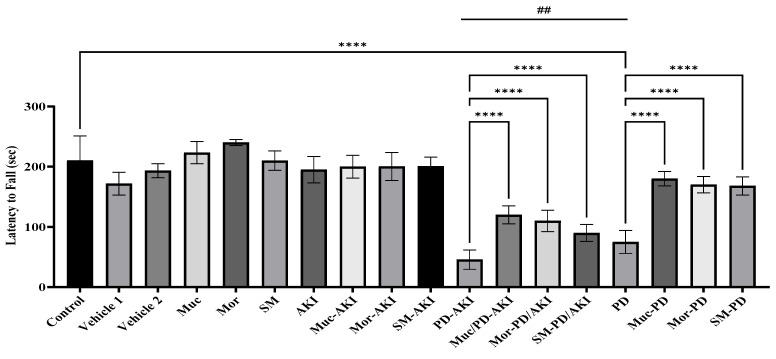
The effect of Muc, Mor, and SM on motor coordination and balance, measured by the rotarod test. The latency to fall (in seconds) was recorded for all the experimental groups. Data are shown as the mean ± SEM (n = 9) and **** *p* < 0.0001. (#) show comparison between PD-AKI vs. PD and ## *p* < 0.01.

In the control group, the mean latency to fall was 210 ± 40.64 s, serving as the baseline for normal neuromotor performance. The vehicle groups showed a mild reduction (171–193 s), while the extract groups (Muc, Mor, and SM) displayed slightly improved latencies (223–240 s), suggesting no adverse and possibly beneficial effects on motor coordination (Figure 13). In the AKI model, the latency decreased moderately to 195 ± 21.79 s compared to the control, without any statistical significance. Pre-treatment with the extracts preserved latencies near baseline (200 s). The dual PD–AKI model produced the most severe impairment, reducing latency to 46 ± 16.11 s, a 4.6-fold decrease compared to the control (*p* < 0.0001). However, the extract pre-treatment conferred strong neuroprotection, where in Muc–PD–AKI, Mor–PD–AKI, and SM–PD–AKI, the latencies were restored 2.6-, 2.4-, and 1.9-fold, respectively, compared to the untreated PD–AKI mice. By contrast, the PD model caused a marked reduction in latency to 75 ± 19.09 s (2.3-fold, *p* < 0.0001), confirming rotenone-induced neuro-motor deficits. The co-treatment with the extracts significantly rescued performance. The Muc–PD, Mor–PD, and SM–PD mice exhibited changes in latencies corresponding to a 2.4-, 2.3-, and 2.2-fold (*p* < 0.0001) increase, respectively, showing improvement over the untreated PD group. Motor coordination deteriorated in PD (75 s), and was the worst in PD–AKI (45.6 s, *p* < 0.001), while AKI showed normal values, confirming that neuromotor deficits are PD-driven and magnified by renal comorbidity.

#### 2.5.2. Pole Climb Test: Half and Final Descent

**Figure 14:**
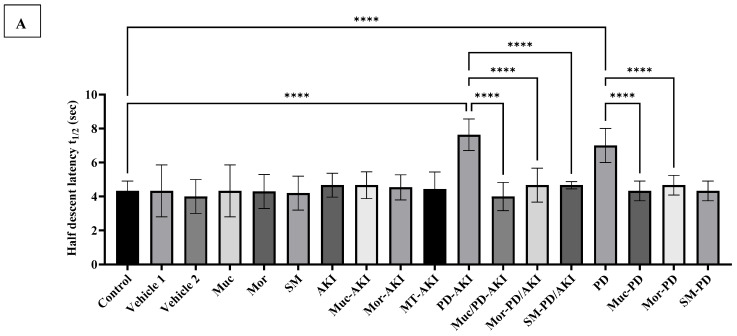

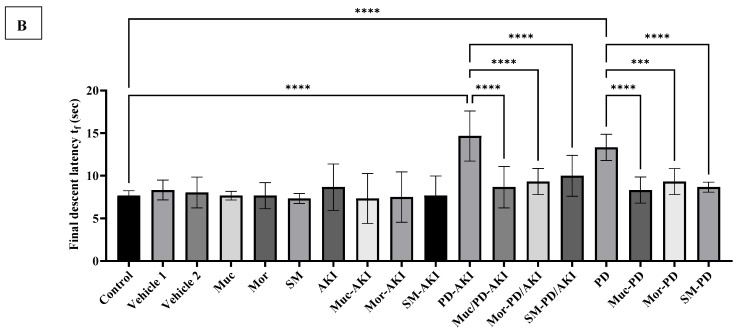
The effect of Muc, Mor, and SM on motor initiation and bradykinesia using the pole climb test. (**A**) shows the half descent latency time (t_1_/_2_, in seconds), and (**B**) shows the full time to descend (t_f_). Data are presented as the mean ± SEM (n = 9); *** *p* < 0.001, and **** *p* < 0.0001.

The pole climb test revealed distinct differences in motor performance across the experimental groups. The control group demonstrated a mean half latency of 4.33 ± 0.57 s (Figure 14A). The vehicle 1, vehicle 2, and Muc, Mor, and SM treatment groups showed normal values similar to the control. The AKI mice and pre-treated mice with the Muc, Mor, or SM extracts did not show noticeable changes in their motor performance compared to the control group. The PD-AKI and PD groups showed markedly prolonged latencies by 1.8-fold and 1.6-fold, respectively, compared to the control group, confirming significant neuromuscular delay due to PD pathology. The Muc/PD-AKI, SM/PD-AKI, and Mor/PD-AKI groups and Muc-PD, Mor-PD, and SM-PD all scored normal latencies, indicating restoration of motor capacity. As for the mean final descent latency (t_f_), the control group yielded a t_f_ of 7.66 ± 0.5 s. Vehicle 1, vehicle 2, Muc, Mor, and SM showed similar normal values (Figure 14B).

In the AKI disease model, all three extract groups showed no significant variations. Whereas, in the other two disease models, PD-AKI and PD, PD-AKI showed a significant increase in t_f_ of 1.7-fold (*p* < 0.0001) and in the PD group of 1.8-fold (*p* < 0.0001) compared to the control, demonstrating coordination and motor impairment and reflecting significant neurological deterioration. In the PD-AKI and PD models, pre-treatment with the plant extracts showed notable mitigation, and the three extracts in both models scored latencies comparable to the control group, highlighting their potential in preserving or restoring motor coordination in PD pathology.

The t_f_ was significant in PD and PD–AKI, while it remained near control levels in the AKI group, again indicating PD-dependent bradykinesia. PD–AKI consistently exceeds PD in delay (*p* < 0.05), mirroring an additive neuro-motor burden under dual-organ stress.

### 2.6. Histological Analysis of Kidney and Brain Tissues

**Figure 15:**
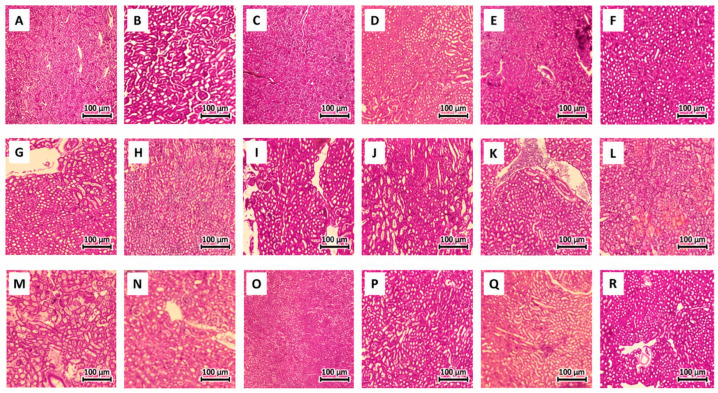
H&E-stained sections of the kidney from all experimental groups, examined under a light microscope (4X & 10X). (**A**) The control group, (**B**) vehicle 1, (**C**) vehicle 2, (**D**) Muc, (**E**) Mor, (**F**) SM, (**G**) AKI, (**H**) Muc-AKI, (**I**) Mor-AKI, (**J**) SM-AKI, (**K**) PD-AKI, (**L**) Muc/PD-AKI, (**M**) Mor/PD-AKI, (**N**) SM/PD-AKI, (**O**) PD, (**P**) Muc-PD, (**Q**) Mor-PD, and (**R**) SM-PD.

Microscopic examination of the kidneys of the control mice showed typical renal architecture, with normal glomeruli and normal proximal and distal tubules with normal interstitial cells, as shown in Figure 15A. The kidneys of the mice treated with the SM, Muc, and Mor extracts, as well as the vehicles, showed no histological changes and were similar to normal kidneys (Figure 15B–F). However, the kidneys of the mice with APAP administration showed normal glomeruli and a slight rise in interstitial mesenchymal cells (Figure 15G). Administration of Muc, Mor, or SM alone did not produce any histological alterations, and kidney morphology remained comparable to that of the normal control group (Figure 15H–J). Additionally, the kidneys of the PD-AKI mice showed normal glomeruli and tubules, with marked mixed inflammatory cell infiltration in the medulla (Figure 15K). Also, the pre-treatment groups Muc/PD-AKI and Mor/PD-AKI showed normal glomeruli, with mild swelling of the tubular-lined endothelial cells (Figure 15L,M). On the other hand, the pre-treatment group SM/PD-AKI showed normal kidneys (Figure 15N). PD induction in the mice, as well as the co-treated mice with all three extracts, did not show noticeable histopathological alterations (Figure 15O–R). Structural injury was evident in AKI and PD–AKI (interstitial inflammation/changes), while PD kidneys resembled those of the control, aligning histology with functional and molecular renal injury. Lesion burden appeared greater in PD–AKI than AKI, supporting exacerbation of renal pathology by concomitant PD.

**Figure 16:**
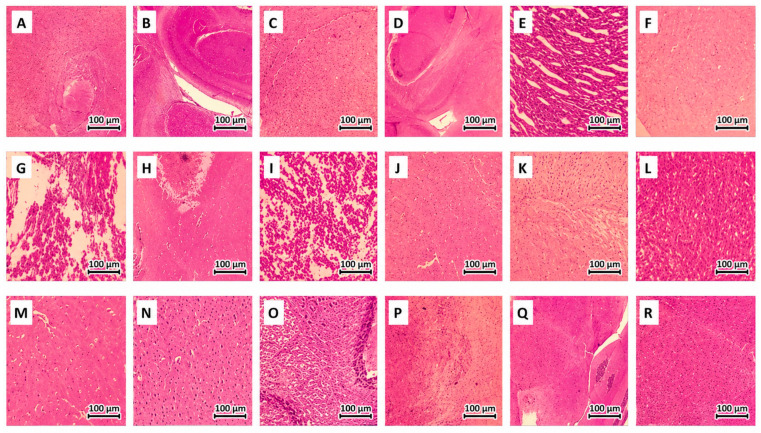
H&E-stained sections of the *brain tissue* from all the experimental groups, examined under a light microscope (4X & 10X). (**A**) The control group, (**B**) vehicle 1, (**C**) vehicle 2, (**D**) Muc, (**E**) Mor, (**F**) SM, (**G**) AKI, (**H**) Muc-AKI, (**I**) Mor-AKI, (**J**) SM-AKI, (**K**) *PD*-AKI, (**L**) Muc-*PD*/AKI, (**M**) Mor-*PD*/AKI, (**N**) SM/PD-AKI, (**O**) PD, (**P**) Muc-PD, (**Q**) Mor-PD, and (**R**) SM-PD.

Histological examination of the brains using H&E staining is shown in Figure 16. The control, vehicle 1, vehicle 2, Muc, Mor, and SM groups (Figure 16A–F) demonstrated normal neuronal architecture, characterized by intact cells and absence of degeneration signs, showing normal color of the neural cells in the brain substantia nigra. However, the brain of the AKI mice showed mild gliosis and depigmentation of the substantia nigra (Figure 16G). On the other hand, the pre-treatment with the Muc extract before AKI induction preserved normal brain architecture, revealing neuroprotective potentials (Figure 16H). The SM-AKI mice did not show any gliosis but still showed depigmentation of the substantia nigra, revealing limited protection (Figure 16J). However, Mor-AKI did not show any enhancement in the brain architecture (Figure 16I). The PD-AKI group (Figure 16K) exhibited noticeable gliosis and depigmentation of the substantia nigra. But, Muc/PD-AKI (Figure 16L) and SM/PD-AKI (Figure 16N) restored normal brain architecture, revealing neuroprotective potentials. The Mor/PD-AKI (Figure 16M) group showed partial gliosis and depigmentation of the substantia nigra, revealing limited recovery in the brain architecture. The PD group showed moderate gliosis and depigmentation of the substantia nigra (Figure 16O). Muc-PD showed no histological changes and was comparable to the normal brain (Figure 16P). Mor-PD and SM-PD showed mild gliosis (Figure 16Q,R). Gliosis and substantia nigra depigmentation were observed in PD and amplified in PD–AKI, whereas the AKI group showed only partial changes, consistent with PD-centric neurodegeneration. The PD–AKI > PD > AKI histological gradient matches the oxidative/inflammatory and behavioral gradients observed.

## 3. Discussion

In a murine model of AKI, PD, and their co-morbidity (PD-AKI), this investigation presents preliminary preclinical evidence supporting the nephroprotective and neuroprotective attributes of Muc, Mor, and SM extracts. By integrating functional, biochemical, molecular, behavioral, and histopathological analyses, we demonstrate that all three extracts exerted nephroprotective and neuroprotective effects, primarily through attenuation of oxidative stress, suppression of inflammatory mediators, or restoration of PI3K/AKT/mTOR signaling.

Different studies were conducted to assess the safety profile of the three plants. Muc showed no cytotoxicity in human cervical adenocarcinoma (HeLa) cells at 50 µg/mL aqueous extract, indicating a satisfactory biocompatibility profile [44]. A multi-herbal mixture including Muc (25 mg), delivered intraperitoneally to mice for a duration of 6 to 8 weeks, did not elicit detectable toxicity. Notably, the same formulation was documented as safe at a dosage of 5000 mg/kg, further corroborating the plant’s substantial safety margin [45]. These data together confirm that Muc is well-tolerated at both therapeutic and high experimental dosages [46], and the dose we used in this study (350 mg/kg) is considered safe. For the Mor aqueous extract, toxicological assessments indicated a substantial safety margin, as high oral doses (up to 2000 mg/kg) in rats did not induce any severe systemic toxicity [47], reflecting also the safety of our chosen tested dose in this study, i.e., 350 mg/kg. The same holds true for the SM dose used (350 mg/kg), where a toxicological study showed that its acute toxicity starts at 400 mg/kg in mice [48]. These studies, along with our preliminary toxicity assessments, show that the administered dose is well within the experimentally validated safe range, supporting the reliability and translational relevance of our dosing regimen. As for rotenone, the daily intraperitoneal dose used in this study (2.5 mg/kg for 21 days) is consistent with the experimental range that effectively reproduces Parkinsonian pathology, while ensuring the well-being of the animals involved. Multiple studies indicated that systemic exposure to 1–3 mg/kg of rotenone over a period of approximately three weeks leads to a progressive loss of dopaminergic neurons and associated motor deficits, while not resulting in the mortality seen with higher or extended dosing [49,50]. Miyazaki et al. (2020) [51] reported similar outcomes, maintaining mice on 2.5 mg/kg per day via osmotic infusion for four weeks, which resulted in significant nigrostriatal injury, without systemic toxicity. Rocha et al. (2022) [52] observed that splitting the same cumulative dose into bi-daily injections significantly heightened lethality, underscoring the benefits of single daily administration. This evidence collectively indicates that a dosage of 2.5 mg/kg/day for a duration of 21 days is a reliable and ethically sound protocol for simulating Parkinson-like neurodegeneration in murine models.

Consistent with previous AKI models induced by APAP [53], the notable elevation in serum creatinine and BUN levels substantiated the presence of renal dysfunction. The present work also demonstrated a significant upregulation of KIM-1 in both the PD-AKI and AKI models, underscoring its function as a sensitive indicator of renal damage, cell phagocytosis, repair processes, and inflammation [54]. Notably, PD–AKI exhibited greater dysfunction than AKI alone, consistent with the synergistic burden of dual-organ injury. A single high intraperitoneal dose of APAP at 1000 mg/kg is widely recognized to provoke AKI. The toxic metabolite N-acetyl-p-benzoquinone imine (NAPQI) overwhelms the glutathione defense system, leaving renal tubular cells highly vulnerable to oxidative and necrotic damage [55]. Within 24 h, mice typically present with sharp increases in serum creatinine and BUN, accompanied by normal histological features, except for a slight rise in interstitial mesenchymal cells. This slight increase in interstitial mesenchymal cells can explain the early tissue reaction to stress rather than a sign of established fibrosis. In these initial settings, the kidney represents a direct target of APAP toxicity. The PD-AKI model exhibited the worst injury with a significant increase in renal biomarkers (creatinine and BUN), suggesting that neurodegenerative stresses exacerbate renal impairment, aligning with the expanding information concerning the interactions within the brain–kidney axis [9,10]. This synergistic effect provides a strong rationale for expecting the most pronounced renal dysfunction in the comorbid group, where both direct tubular injury and systemic mitochondrial stress converge [55,56]. Pre-treatment with the Muc, Mor, and SM extracts significantly attenuated creatinine and BUN levels, consistent with previous research and indicating that Muc diminishes uremia-associated parameters [57], Mor decreases BUN and creatinine levels [26], and SM alleviates nephrotoxic effects in cisplatin-induced injury models [58]. By contrast, rotenone—administered chronically at low daily doses to reproduce Parkinsonian features—exerts its primary toxicity in the nervous system through inhibition of mitochondrial complex I. Although systemic administration does permit some accumulation in peripheral organs, the renal manifestations are generally secondary. This explains why the PD-only groups (PD, Muc-PD, Mor-PD, and SM-PD) exhibited no significant alterations in renal markers (creatinine and BUN) with normal histological kidney features.

Oxidative stress plays a key role in cellular damage. It is also associated with a direct role in the initiation, development, and progression of AKI and implicated in neurodegeneration occurring in PD. Moreover, an imbalance in the oxidant/antioxidant cell profile accounts for neuroinflammation after the activation of the brain immune cells, the microglia, leading to PD progression [59]. Oxidative stress segregates by organ and model. In the kidney, the antioxidants (SOD and CAT) collapse, and the lipid peroxidation (MDA) rises in AKI and PD–AKI models, while PD mirrors control results. Conversely, in the brain, oxidative injury is PD-driven, where it is accentuated in PD–AKI and is minimal in AKI alone. In the PD/AKI group, the prolonged rotenone treatment established a background of oxidative stress, metabolic exhaustion, and low-grade inflammation. When kidneys under such strain are challenged with APAP, the acute toxic insult is likely to be amplified, as observed in our results.

In our study, both AKI and PD were associated with a notable downregulation of the antioxidant master regulator *Nrf2*, accompanied by an increase in the lipid peroxidation marker MDA and a decrease in the endogenous antioxidant enzyme activities (SOD and CAT). This suggests a disruption in the intrinsic redox balance within both renal and brain tissues, with a notably pronounced severity observed in the PD-AKI model. Significantly, Muc pretreatment was associated with increased *Nrf2* mRNA expression and reactivated the activities of SOD and CAT, concurrently decreasing MDA levels. The results signify an enhancement of both transcriptional and enzymatic antioxidant defenses, attributed to the synergistic actions of phytochemicals—L-DOPA and polyphenols in Muc [44,60]. Attenuation was also observed in Mor and SM, reflecting their established phytochemical antioxidant characteristics due to the quercetin and chlorogenic acid contents in Mor [19,61] and silymarin, the main bioactive component of SM [32,62]. These botanicals presumably function via *Nrf2*-mediated pathways and reactive oxygen species scavenging, as evidenced in our study, and corroborated by recent phytopharmacological studies [12]. This study shows a coherent yet tissue-specific pattern of oxidative stress in the dual PD–AKI model. In the kidney, injury was characterized by a marked reduction in antioxidant defenses (decreased SOD and CAT activities and increased MDA levels). In contrast, the brain exhibited a dysregulated antioxidant response, with increased SOD activity alongside decreased CAT activity and elevated MDA levels, suggesting an imbalance between reactive oxygen species generation and detoxification. Thus, the observed differences reflect tissue-specific and pathway-specific oxidative stress responses. Yet, the underlying mechanisms require more investigation.

In conjunction with the oxidative stress, there was a significant elevation in inflammation observed in the AKI, PD, and PD-AKI models, as indicated by the increased expression of the *NF-κB* mRNA level and the elevated levels of IL-6 and TNF-α in both the renal and brain tissues. Higher renal cytokine levels and increased NF-κB mRNA expression were shown in AKI (maximal in PD–AKI), whereas PD alone was quiescent. Centrally, PD, and especially PD–AKI, drove a robust increase in IL-6/TNF-α and NF-κB mRNA expression, with AKI mirroring the control. The concurrent rise of IL-6 and TNF-α in renal and cerebral tissues across illness models underscores a systemic inflammatory axis, perhaps influenced by cytokine spillover, blood–brain barrier disturbance, or microglial activation. *NF-κB* mRNA expression was significantly higher in the brain of both the PD and PD-AKI groups, with the PD-AKI group showing a substantial induction (3.8-fold, *p* < 0.0001), underscoring the pronounced neuroinflammatory response due to the interplay of neurodegeneration and renal injury. The observed increase in *NF-κB* indicates an enhanced inflammatory environment in the brain, consistent with earlier findings regarding the interaction between systemic inflammation and neurodegenerative disease. In the PD-AKI animals, levels of inflammatory cytokines were elevated, indicating the cumulative consequences of renal and neurological damage. Although not always restoring absolute baseline—particularly in the brain—the extracts reduced cytokine levels and decreased NF-κB mRNA expression across models, with Muc showing the most consistent anti-inflammatory effect and enhancing the antioxidant defense. The observed decrease in *NF-κB* mRNA expression supports this anti-inflammatory impact. Nevertheless, this transcriptional change does not verify direct NF-κB inhibition and should be interpreted as an associative rather than mechanistic finding.

Oxidative stress triggers neuronal cell death, playing a significant role in the pathogenesis of PD [63] and AKI [64]. The PI3K-AKT pathway controls oxidative stress by affecting molecular targets, such as mTOR [65]. In this study, the PI3K, AKT, and mTOR mRNA expression levels decreased in the AKI, PD, and AKI-PD models. Researchers have found that AKT levels are markedly reduced in individuals with PD [66], corroborating our results in the PD and PD-AKI models. This downregulation also extended to mTOR gene expression, contributing to neurodegeneration [65].

As for the AKI models, our study is the first to evaluate the impact of APAP-induced AKI on PI3K/AKT/mTOR signaling. While direct evidence is limited, studies have shown the involvement of this pathway and its downregulation in kidney injury, in the absence or presence of diverse nephrotoxins [67,68] or lipopolysaccharide [69]. In the present study, reduced mRNA expression of PI3K, AKT, and mTOR was observed, with the most pronounced expression in the PD-AKI brain tissue, indicating a failure of neuronal resilience under dual-organ stress. In the kidney, AKI—particularly in the PD–AKI condition—was associated with the decreased mRNA expression of PI3K/AKT/mTOR components, but PD alone remained near baseline. However, in the brain, PD was associated with reduced expression of these genes, with a more marked decrease in the PD–AKI model, and AKI alone had little effect. All three extracts partially rescued the pathway expression in both organs, with Muc yielding the largest and most consistent recovery, indicating convergent protection across redox, inflammatory, and pro-survival nodes. The capacity of Muc, and to a lesser degree Mor and SM, to partly reinstate this pathway gene expression highlights the pharmacodynamic potential of these extracts in reactivating pro-survival signaling. Yet, as these results are based on transcriptional data, they do not confirm direct activation or restoration of the PI3K/AKT/mTOR signaling pathway.

A comprehensive battery of behavioral tests revealed that PD [70], not AKI, was the primary driver of functional impairments. Rotarod performance and pole test latencies all confirmed profound motor, reflex, and coordination deficits in the PD and PD-AKI mice. Pretreatment with the three extracts provided complete protection against PD-induced deficits. This could be due to the presence of their active secondary metabolites. For instance, Muc is widely recognized for its high L-DOPA content, which likely supports dopaminergic tone and synaptic resilience.

The worst effect being observed in PD-AKI models without extract treatment suggests that renal insult compounds CNS stress. This is most likely driven by systemic inflammation, uremic toxin build-up, and impaired mitochondrial bioenergetics—pathways well-documented in PD-AKI associations [71,72,73]. Yet, these mechanisms were not directly assessed in the present study.

The efficacy of Muc, Mor, and SM extracts to restore antioxidant defenses, inhibit cytokines, and restore motor function demonstrates phytochemicals’ therapeutic promise in neurodegenerative and renal ailments. The findings support the rise of phytotherapy approaches that utilize multi-target interventions, employing plant secondary metabolites to modulate oxidative, inflammatory, and apoptotic pathways [74]. Our study lacks the comprehensive phytochemical characterization of the plant extracts; this limitation may affect the interpretation of the results, as the observed biological effects cannot be attributed to specific molecules or mechanisms. Thus, future investigations incorporating detailed phytochemical profiling and standardization are important to better define the active constituents and improve reproducibility and translational relevance.

## 4. Materials and Methods

### 4.1. Preparation of Aqueous Plant Extracts

Muc (krounchbeej) seed powder was bought from Royal Herbal Land Pvt. Ltd. (Pune-410401, Maharashtra, India). Mor (the young shoot and total aerial part) and SM (seeds only) were grown in Nabatieh-Deir El Zahrani, Lebanon. They were provided and authenticated by Dr. Mohammad Tarabulsi (Ph.D. in Herbology and Homeopathy). The plants included in this study were chosen based on evidence reported in the literature demonstrating their significant biological properties, including antioxidant, anti-inflammatory, and therapeutic effects. In general, Muc is rich in L-DOPA and other alkaloids, Mor contains a wide range of phenolic compounds and flavonoids, while SM is characterized by the presence of silymarin and other phenolic compounds (Supplementary File S1 Tables S1–S3) [15,75,76,77,78,79,80,81,82] The plants were collected, washed, and dried in a clean, well-ventilated, dark environment. Following drying, the plants were ground and pulverized. The plant powders were stored in a cool, dry, dark environment with controlled humidity levels to prevent moisture-related degradation for a period not exceeding six months. A 3% aqueous solution of each plant or seed was prepared by boiling 3 g in 100 mL of distilled water. The temperature was precisely maintained at 100 °C for 30 min. Extraction was followed by centrifugation at 6000 rpm for 10 min. The supernatants underwent sequential filtration, first through sterile gauze to remove larger particles, followed by filtration through Whatman filter paper for finer purification. The final filtrates were distributed among sterile containers and preserved at −20 °C to maintain their stability and bioactivity for future experiments.

### 4.2. Animals

Male BALB/c mice weighing 20 ± 5 g were obtained from the Beirut Arab University animal facility. The experimental procedure was approved by the Institutional Review Board (IRB) of Beirut Arab University, code number (IRB 2023-A-0052-S-M-0553) on 13 December 2023, abiding by the ARRIVE guidelines and the Canadian Council on Animal Care’s (CCAC) guides for experimental animals’ usage and caring. All efforts were made to minimize animal suffering, and humane endpoints were applied in accordance with institutional guidelines. Also, all experiments were performed by a trained person, according to the institutional animal care guidelines. The mice were kept under standard laboratory settings of light (12 h light/dark cycle), adequate room temperature, and humidity, with free feeding following a standard mouse diet and water. The mice were left to acclimate for one week before beginning the experiment.

### 4.3. Experimental Design: AKI and PD Induction

AKI was induced in the mice by a single intraperitoneal (i.p.) dose of APAP suspended in 10% DMSO at 1000 mg/kg body weight [83]. aqueous extract was given orally at 350 mg/kg for 21 days before AKI induction. This dose was selected based on previous publications [30,46,47]. A preliminary study was conducted to determine the efficiency of the APAP concentration in inducing AKI by measuring the kidney function parameters and histological studies. A modified version of the Rocha et al. (2022) protocol [52], with an extended length of 21 days, was utilized to generate PD and to elicit more pronounced Parkinsonian symptoms similar to those found in PD patients. The mice were administered an intraperitoneal injection of rotenone, a frequently utilized chemical for inducing PD, at a dosage of 2.5 mg/kg/day for a consecutive period of 21 days. The rotenone was prepared daily in DMSO at a final concentration of 10 mg/mL and subsequently diluted in sesame oil before application. The rotenone stock was filtered using 0.22 µm membrane filters before dilution in sesame oil under aseptic conditions.

A total of 18 groups, each including 6 mice (total n = 108), were used as indicated in Table 1. The sample size was calculated with G*Power (v3.1) (effect size f = 0.50 (Cohen’s f), α = 0.05, power (1 − β) = 0.80, number of groups = 18). For biochemical and molecular analyses, n = 3–6 biological replicates were analyzed per group, while behavioral tests included n = 9 animals per group.

Treatment with each extract was also given by gavage daily over 21 days at a concentration of 350 mg/kg b.w., concomitant with PD induction. Preliminary toxicity assessments were conducted to determine the safety of the extracts, which were determined through observable behavioral or physiological changes and evidenced by normal kidney function and histology. For PD and AKI induction and treatment, the mice were intraperitoneally (i.p) injected with rotenone 2.5 mg/kg daily over successive 21 days, with or without extract treatment. After 24 h from the last induction–treatment period, AKI was induced by a single i.p. injection of APAP.

The mice’s body weight was properly monitored daily before receiving the exact amount of treatment. At the end of the experimental study, all the mice were anesthetized by intraperitoneal injection of a mixture of ketamine (50 mg/Kg) and xylazine (10 mg/Kg), followed by cervical dislocation. Twenty-four hours after the last treatment, blood samples were collected through cardiac puncture to determine creatinine and urea levels, and the animals were dissected, with the brains and kidneys dissected out and washed with phosphate buffer saline. The organs were then immediately collected and either fixed with 10% formaldehyde in phosphate buffer (pH = 7.4) or frozen in liquid nitrogen, and then stored at −80 °C for later use.

Any mouse revealing any inflammatory signs leading to biased results was excluded from this study. Less than 1% of the total mice were lost due to mouse movement during the intraperitoneal injection of rotenone or APAP, causing accidental puncture of organs in the abdomen, leading to internal bleeding and immediate death of the animal. This was observed upon dissecting the animal directly after death. To reduce confounding factors, each time, all procedures were carried out by the same researcher, and the mice were returned to the same place in their cages. Table 1 summarizes the treatment of the different experimental groups.

### 4.4. Histological Analysis

Both kidney and brain tissues were fixed in 10% formaldehyde. Kidney tissue block sections of 5 µm thickness and brain tissue block sections of 6 µm thickness were prepared. The slides were stained by Hematoxylin and Eosin (H&E) and examined under a Zeiss Primo light microscope (Carl Zeiss, Oberkochen, Germany) connected to an AxioCam camera (Carl Zeiss, Oberkochen, Germany) at 4x and 10x magnification.

### 4.5. Determination of Antioxidant Activities and Levels

The kidneys and brain were homogenized in lysis buffer consisting of 10 mM phosphate buffer saline (PBS), with 1 mM phenyl methyl sulfonyl fluoride (PMSF) at a 1:9 ratio (*w*/*v*), followed by centrifugation for 5 min at 6000× *g*. The supernatant was collected for analysis. Levels of malondialdehyde content (MDA), catalase (CAT), and superoxide dismutase (SOD) were determined according to Buege JA and Aust SD (1978) [84].

### 4.6. Cytokine Quantification

IL-6 and TNF-α were quantified using ELISA kits, E-ELM0044 Mouse IL-6 and E-EL-M3063 Mouse TNF-α, purchased from Elabscience (Wuhan, China), according to the manufacturer’s protocol.

### 4.7. Gene Expression Analysis by Quantitative Real-Time PCR (qPCR)

Total RNA was extracted from brain tissue using the RNeasy Mini Kit (Qiagen, Hilden, Germany), according to the manufacturer’s protocol. The purity and concentration of the isolated RNA were assessed spectrophotometrically by measuring absorbance at 260/280 nm, using a NanoDrop™ spectrophotometer (Thermo Fisher Scientific, Waltham, MA, USA). As for the kidney tissue, RNA extraction was accomplished using TRIzol reagent (Invitrogen, Carlsbad, CA, USA), as suggested by the manufacturer. The RNA was quantified and used for the reverse transcriptase reaction, using the QuantiTect Reverse Transcription Kit (Qiagen, Germany). Gene quantification was performed using qRT-PCR, based on the primers listed in Table 2. Relative gene expression levels of *Nrf2*, *NF-κB*, *PI3K*, *AKT*, *mTOR*, and *KIM-1* were calculated using the comparative ΔΔCt method [85], with GAPDH used as the internal housekeeping control gene. All reactions were performed in triplicate, and the results were expressed as fold changes relative to the untreated control group.

### 4.8. Behavioral Study: Pole Climbs and Rotarod Tests

Blinded behavioral studies were conducted. Baseline assessments were conducted at time zero to confirm the uniformity of all the mice at the start of the experiment. Behavioral assessments were conducted at 21 days (the end of the co-treatment phase) to evaluate changes in each mouse’s behavior, which serves as a significant indicator of both the induction of PD and the effects of treatment. In addition, behavioral assessments were conducted after AKI induction. The mice were allowed to acclimate to the testing environment before beginning the test. The duration and frequency of testing were minimized to reduce stress.

Motor dysfunction was evaluated by pole climb and rotarod tests, as described in [86]. For both tests, the mice were trained before the experiment. For the pole climb test, the time the mouse needed to cross half the pole length and the total length were recorded. The mice were positioned on a 70 cm vertical pole that was 1 cm in diameter. To encourage the mice to drop to the cage floor, the pole was positioned in the home cage and fixed on a rectangular base stand. The experiment was repeated if the animal hesitated while descending. The pole’s surface was taped to prevent it from slipping. Each animal underwent the test three times. The rotarod test was set with a starting speed of 4 rpm and hastened to reach 40 rpm gradually for 300 s. The maximum time the mouse remained on the rod, which was proportional to the latency to fall, was recorded. Each animal underwent the test three times.

### 4.9. Statistical Analysis

Statistical analysis was performed using GraphPad Prism 10.4.1. All experimental results were analyzed by one-way ANOVA, followed by Tukey’s test. All results were expressed as the mean value ± SEM. A level of *p* ≤ 0.05 was considered statistically significant. Data distribution was assessed for normality prior to parametric testing. The sample size (n = 3 per group) was selected based on the exploratory nature of this study and in accordance with ethical guidelines to minimize animal use, following the principles of the 3Rs (Replacement, Reduction, and Refinement).

## 5. Conclusions

Our investigation reveals that Muc, Mor, and SM are associated with nephroprotective and neuroprotective effects in AKI, PD, and AKI-PD murine models. Together, the observed patterns within the obtained results support a bidirectional brain–kidney axis, where the dual pathology (PD–AKI) imposes additive or synergistic oxidative, inflammatory, and signaling burdens, and where phytoprophylaxis exerts multimodal protection rather than a single-pathway effect. By utilizing a comprehensive analytical approach, we have demonstrated that these extracts significantly rejuvenate renal function, boost antioxidant mechanisms, mitigate inflammatory responses, alter the mRNA expression of pathways related to cell survival, and enhance both motor and cognitive capabilities. Our results emphasize the potential therapeutic applications of these botanicals in addressing the complexities of coexisting renal and neurodegenerative conditions, while also illuminating the significant interactions between kidney and brain pathologies. Still, advanced studies incorporating protein-level validation, pathway-specific functional assays, and clinically relevant models are essential to clarify the underlying mechanisms and evaluate translational applicability.

Although the current investigation provides substantial evidence supporting the protective properties of the three extracts, several limitations must be acknowledged. First, while various parameters were measured and assessed, the investigation was constrained to acute disease models. Chronic or progressive models could uncover distinct or more intricate therapeutic dynamics, especially regarding neurodegeneration. Secondly, the specific active components and their mechanisms of action for each herbal extract are not fully elucidated. The observed effects are likely attributed to the known antioxidant and anti-inflammatory compounds present (e.g., L-DOPA, quercetin, and silibinin). However, comprehensive phytochemical profiling and pathway–target validation, such as through inhibitor studies or knockouts, are essential to accurately identify their molecular targets and interactions and to establish direct causal relationships. Furthermore, this investigation employed preventive strategies before treatment, thereby constraining direct application to therapeutic scenarios. Future investigations ought to examine the post-injury application of these extracts to determine their potential to remediate existing damage. Further studies are required to validate these findings in more clinically relevant models and to assess their translational potential in human subjects.

## Figures and Tables

**Table 1:** Experimental groups and treatment protocols for AKI, AKI-PD, and PD mouse models.

Group	Description
Control	Normal mice; negative control group.
Vehicle 1	Normal mice were i.p. injected with DMSO in water (vehicle for APAP).
Vehicle 2	Normal mice were injected with the vehicle used to dissolve rotenone.
Muc	Normal mice were given Muc for 21 days.
Mor	Normal mice were given Mor for 21 days.
SM	Normal mice were given SM for 21 days.
AKI	Mice were i.p. injected with a single dose of APAP (1000 mg/kg) to induce AKI.
Muc-AKI	Mice were pretreated with Muc for 21 days, then given APAP to induce AKI.
Mor-AKI	Mice were pretreated with Mor for 21 days, then given APAP to induce AKI.
SM-AKI	Mice were pretreated with SM for 21 days, then given APAP to induce AKI.
PD	Mice were pretreated with rotenone for 21 days to induce PD.
Muc-PD	Mice were co-treated with rotenone and Muc daily for 21 days.
Mor-PD	Mice were co-treated with rotenone and Mor daily for 21 days.
SM-PD	Mice were co-treated with rotenone and SM daily for 21 days.
PD-AKI	Mice treated with rotenone for 21 days, then given APAP to induce AKI.
Muc\PD-AKI	Mice treated with rotenone + Muc for 21 days, then given APAP to induce AKI.
Mor\PD-AKI	Mice treated with rotenone + Mor for 21 days, then given APAP to induce AKI.
SM\PD-AKI	Mice treated with rotenone + SM for 21 days, then given APAP to induce AKI.

**Table 2:** Primer sequences (forward and reverse) of the studied genes.

Primer	Forward (5’–3’)	Reverse (5’–3’)	Accession Number	Reference
*NF-κB*	GAAATTCCTGATCCAGACAAAAAC	ATCACTTCAATGGCCTGTGTGTAG	NM_009045.5	NCBI (RefSeq); Primer-BLAST
*NrF2*	CAGCATAGAGCAGGACATGGAG	GAACAGCGGTAGTATCAGCCAG	NM_010902.4	NCBI (RefSeq); Primer-BLAST
*mTOR*	AGAAGGGTCTCCAAGGACGACT	GCAGGACACAAAGGCAGCATTG	NM_020009.2	NCBI (RefSeq); Primer-BLAST
*PI3K*	ATCATGCAAATCCAGTGCAA	CAGCTGTCCGTCATCTTTCA	NM_008839.3	NCBI (RefSeq); Primer-BLAST
*AKT*	ATCCCCTCAACAACTTCTCAGT	CTTCCGTCCACTCTTCTCTTTC	NM_009652.3	NCBI (RefSeq); Primer-BLAST
*KIM-1*	ACATATCGTGGAATCACAACGAC	ACTGCTCTTCTGATAGGTGACA	NM_134248.2	NCBI (RefSeq); Primer-BLAST
*GAPDH*	CATCACTGCCACCCAGAAGACTG	ATGCCAGTGAGCTTCCCGTTCAG	NM_008084.3	NCBI (RefSeq); Primer-BLAST

Primers were designed using Primer-BLAST (NCBI, Bethesda, MD, USA; https://www.ncbi.nlm.nih.gov/tools/primer-blast/ (accessed on 26 April 2026).

## Data Availability

All data that support our findings in this study are available from the corresponding author (Jamilah Borjac) upon reasonable request.

## Supplementary Materials

The following supporting information can be downloaded at: https://www.mdpi.com/article/10.3390/ijms27094021/s1.

## Abbreviations

The following abbreviations are used in this manuscript:

Muc	*Mucuna pruriens*
Mor	*Moringa oleifera*
SM	*Silybum marianum* (Milk Thistle)
PD	Parkinson’s Disease
AKI	Acute Kidney Injury
PD-AKI	Comorbid Parkinson’s Disease and Acute Kidney Injury
*PI3K*	Phosphoinositide 3-kinase
*AKT*	Protein Kinase B
*mTOR*	Mammalian Target of Rapamycin
*Nrf2*	Nuclear factor erythroid 2-related factor 2
*NF-κB*	Nuclear Factor kappa-light-chain-enhancer of activated B cells
qRT-PCR	Quantitative Real-Time Polymerase Chain Reaction
mRNA	Messenger Ribonucleic Acid
KIM-1	Kidney Injury Molecule-1
α-syn	Alpha-synuclein
L-DOPA	L-3,4-dihydroxyphenylalanine
ROS	Reactive Oxygen Species
BUN	Blood Urea Nitrogen
SOD	Superoxide Dismutase
CAT	Catalase
MDA	Malondialdehyde
IL-6	Interleukin-6
TNF-α	Tumor Necrosis Factor-alpha
APAP	Acetaminophen (N-acetyl-p-aminophenol)
SEM	Standard Error of the Mean
H&E	Hematoxylin and Eosin
