# Supplementary material for: Dual Neuroprotective and Nephroprotective Effects of Mucuna pruriens, Moringa oleifera, and Silybum marianum (Milk Thistle) via Modulation of PI3K/AKT/mTOR and Nrf2/NF-κB Pathways in a Murine Comorbid PD–AKI Model
Source: Int J Mol Sci. 2026 Apr 30;27(9):4021. doi: 10.3390/ijms27094021 (PMC13163383; doi:10.3390/ijms27094021)
Supplement: Supplementary file 1 [file ijms-27-04021-s001.zip › ijms-4254197-supplementary.pdf]

Table S1. The chemical composition of aqueous extracts for *Moringa oleifera* (Mor)

| Class | Representative compounds | Analytical method | ref |
|---|---|---|---|
| Phenolic acids | Chlorogenic acid, caffeic acid | HPLC | 1-2 |
| Flavonoids | Quercetin, kaempferol, rutin | HPLC, UV-Vis | 2 |
| Tannins | Hydrolysable and condensed tannins | Colorimetric assays | 3 |
| Saponins | Triterpenoid saponins | Phytochemical screening | 3 |
| Alkaloids | Various nitrogenous bases | Phytochemical screening | 3 |
| Glycosides | Cardiac glycosides | Qualitative tests | 3 |
| Carbohydrates | Glucose, polysaccharides | GC-MS, HPLC | 1 |
| Proteins/ amino acids | Essential amino acids | Kjedahl/ amino acid analysis | 5 |
| Organic acids | Malic acid, citric acid | GC-MS | 4 |
| Minerals | Ca, K, Fe | AAS/ ICP | 3 |

References:

1-Feng Y, et al. (2025). Composition and anti-colitis efficacy of aqueous extract of Moringa oleifera leaves. Fitoterapia.

2-Azraida H, et al. (2025). Chemical composition and antioxidant activity of aqueous extract of Moringa oleifera. Natural Product Research.

3-Ahmed M, et al. (2023). Proximate composition and antimicrobial activity of Moringa oleifera leaf extracts. International Journal of Molecular Sciences.

4-Khan W, et al. (2017). Metabolomic profiling of Moringa oleifera leaf extract using GC–MS. Frontiers in Pharmacology.

5-Madi N, et al. (2016). Moringa oleifera's Nutritious Aqueous Leaf Extract Has Anticancerous Effects by Compromising Mitochondrial Viability in an ROS-Dependent Manner. Journal of the American College of Nutrition.

Table S2. The chemical composition of aqueous extracts for *Mucuna pruriens* (Muc)

| Class | Representative compounds | Analytical method | ref |
|---|---|---|---|
| Non-protein amino acids | L-DOPA (L-3,4-dihydroxyphenylalanine) | HPLC, LC-MS | 1 |
| Amino acids | Alanine, arginine, glumatic acid, leucine, valine | HPLC, amino acid analyzer | 1 |
| Phenolic acids | Gallic acid, caffeic acid, ferulic acid | HPLC, Folin-Ciocalteu assay | 2 |
| Flavonoids | Quercetin, kaempferol derivatives | HPLC, UV-Vis spectrophotometry | 2 |
| Alkaloids/ indole compounds | Serotonin, tryptamine, DMT, bufotenine | LC-MS, GC-MS | 1 |
| Tannins | Polymeric phenolics | Vanillin assay, Folin-Ciocalteu assay | 2 |
| Saponins | Triterpenoid saponins | Frothing test, spectrophotometric assays | 2 |
| Fatty acids | Linoleic, arachidic, behenic acid | GC-MS | 1 |
| Carbohydrates | Raffinose, oligosaccharides | HPLC, GC-MS | 2 |

References:

1-Kamkaen, N., Chittasupho, C., Vorarat, S., Tadtong, S., Phrompittayarat, W., Okonogi, S., & Kwankhao, P. (2022). Mucuna pruriens seed aqueous extract improved neuroprotective and acetylcholinesterase inhibitory effects compared with synthetic L-Dopa. Molecules, 27(10), 3131. https://doi.org/10.3390/molecules27103131

2-A comprehensive review of the therapeutic potential of Mucuna pruriens. (2026). Journal details pending. https://pubmed.ncbi.nlm.nih.gov/41828855

Table S3. The chemical composition of aqueous extracts for *Silybum marianum* (SM)

| Class | Representative compounds | Analytical method | ref |
|---|---|---|---|
| Flavonolignans (silymarin complex) | Silybnin, silychristin, silydianin | HPLC, LC-MS | 1,2 |
| Phenolic acids | Caffeic acid, chlorogenic acid, ferulic acid | HPLC, Folin-Ciocalteu assay | 1 |
| Flavonoids | Taxifolin, quercetin | HPLC, UV-Vis spectrophotometry | 2 |
| Tanins | Polyphenolic compounds | Spectrophotometric assays | 1 |
| Carbohydrates | Polysaccharides , soluble sugars | HPLC, GC-MS | 1 |
| Lipids | Linoleic acid , oleic acid | GS-MS | 2 |
| Minerals | K,Ca,Mg,Fe | AAS, ICP-OES | 1 |

References:

1-Abenavoli, L., Izzo, A. A., Milic, N., Cicala, C., Santini, A., & Capasso, R. (2018). Milk thistle (Silybum marianum): A concise overview on its chemistry, pharmacological, and nutraceutical uses in liver diseases. Phytotherapy Research, 32(11), 2202–2213. https://doi.org/10.1002/ptr.6171

2-Kidd, P. M., & Head, K. (2005). A review of the bioavailability and clinical efficacy of milk thistle phytosome: A silybin–phosphatidylcholine complex (Silybum marianum). Alternative Medicine Review, 10(3), 193–203.
